# RpoS impacts global gene expression and carbon source utilization in *Escherichia coli* O104:H4

**DOI:** 10.3389/fmicb.2025.1758449

**Published:** 2026-01-20

**Authors:** Petya Berger, Karla Bosse-Plois, Wolfgang Pölking, David Loewe, Ian U. Kouzel, Michael Berger, Ulrich Dobrindt, Alexander Mellmann

**Affiliations:** 1 Institute of Hygiene, University of Münster, Münster, Germany; 2 National Consulting Laboratory for Hemolytic Uremic Syndrome, Institute of Hygiene, University of Münster, Münster, Germany; 3 Department of Biology, University of Konstanz, Konstanz, Germany

**Keywords:** *E. coli* O104:H4, RpoS, global gene expression, metabolism, carbon source utilization, bacterial growth and competition

## Abstract

**Background:**

*Escherichia coli* (*E. coli*) O104:H4 caused the 2011 enterohemorrhagic *E. coli* (EHEC) outbreak in Germany, which remains the outbreak with the highest incidence of hemolytic uremic syndrome worldwide. We recently identified an *E. coli* O104:H4 isolate carrying a single nucleotide polymorphism in the start codon (ATG > ATA) of *rpoS*, which encodes the alternative sigma factor RpoS, resulting in reduced RpoS levels and enhanced virulence gene expression.

**Methods and results:**

Gene set enrichment analysis further revealed that the *rpoS* ATG > ATA mutation was primarily associated with activation of numerous metabolic pathways and repression of carbon source utilization-related transporter and transcription factor genes. Consistently, BIOLOG phenotype microarrays showed that *E. coli* O104:H4 *rpoS* ATG > ATA assimilated amino acids and organic acids (TCA cycle substrates) more efficiently, whereas the wild type strain displayed stronger metabolic respiration with sugars and sugar derivatives, including constituents of the mucus. Deletion of *rpoS* (Δ*rpoS*) in *E. coli* O104:H4 Δ*stx2* resulted in a carbon source utilization profile similar to the one of *rpoS* ATG > ATA, as well as in enhanced growth in minimal medium supplemented with amino acids and reduced one with sugars. Moreover, co-culture experiments with *E. coli* O104:H4 Δ*stx2* and *E. coli* O104:H4 Δ*stx2* Δ*rpoS* revealed a strong competitive advantage of the Δ*rpoS* strain with the tested amino acids; however, no advantage of the *rpoS*-intact strain was observed with sugars.

**Conclusion:**

Our findings elucidate the impact of RpoS on global gene expression and carbon source utilization in *E. coli* O104:H4, further underscoring its role as a central regulator in pathogenic bacteria.

## Introduction

*Escherichia coli* (*E. coli*) is an integral constituent of the mammalian gut microbiota (Tenaillon et al., 2010). However, certain *E. coli* strains can cause various intestinal and extra-intestinal diseases in humans. A prominent example is enterohemorrhagic *E. coli* (EHEC), an intestinal pathogen that can cause hemorrhagic colitis and lead to the life-threatening hemolytic uremic syndrome (HUS) (Karch et al., 2005). The cardinal virulence factor of EHEC is Shiga toxin (Stx), a potent toxin that causes cell death by irreversibly inhibiting eukaryotic protein synthesis (Melton-Celsa, 2014). The majority of EHEC-associated HUS are attributed to the *E. coli* serotype O157:H7, although non-O157 serogroups have been increasingly recognized as clinically important (Tarr et al., 2005; Johnson et al., 2006). Moreover, strains that combine virulence traits of EHEC and other *E. coli* pathotypes have been associated with more severe clinical outcomes (Santos et al., 2020). Notably, the largest EHEC outbreak in German history and the highest incidence of EHEC-associated HUS worldwide was caused in 2011 by *E. coli* O104:H4, a hybrid strain of EHEC and enteroaggregative *E. coli* (EAEC) (Frank et al., 2011; Mellmann et al., 2011). The scale and severity of the 2011 outbreak—nearly 4,000 reported gastroenteritis cases, over 850 cases of HUS and 54 deaths—combined with the lack of causative treatment for EHEC infections (Kampmeier et al., 2018), underscores the importance of understanding the factors and regulatory mechanisms underlying the exceptional virulence of *E. coli* O104:H4.

Besides RNA polymerase (RNAP) sigma 70, RpoD, the essential sigma factor involved in housekeeping gene expression, bacteria harbor alternative sigma factors that transcribe specific sets of genes in response to particular stimuli (Paget, 2015). The alternative RNAP sigma 38, RpoS, is regarded as a master regulator of adaptation and fitness in gram-negative bacteria, playing a crucial role in diverse cellular processes, e.g., oxidative, acid and osmotic stress resistance, nutrient scavenging (metabolism), and virulence gene expression (Dong et al., 2008a). RpoS both activates and represses transcription and regulates conserved pathways/phenotypes in a highly variable and species-dependent manner (Schellhorn, 2020). For example, the effect of RpoS in the typical EHEC strain *E. coli* O157:H7 EDL933 on global gene expression was found to be substantially different from that in commensal *E. coli* K-12 (Dong and Schellhorn, 2009). The versatile effects of RpoS on gene expression might be explained by the fact that *rpoS* is highly polymorphic in both commensal and pathogenic *E. coli* and a trade-off between high stress resistance (RpoS+) and more efficient nutrient scavenging (RpoS-) is believed to drive the acquisition of *rpoS* mutations (Ferenci, 2003; King et al., 2004).

We recently identified RpoS as a global repressor of virulence gene expression in *E. coli* O104:H4 and typical EAEC (Berger et al., 2024). We analyzed an *E. coli* O104:H4 isolate that had acquired a single nucleotide polymorphism (SNP) in the start codon (ATG > ATA) of *rpoS* during laboratory cultivation. The *E. coli* O104:H4 *rpoS* ATG > ATA strain contained up to a 5-fold lower amount of RpoS and an enhanced expression of EAEC-specific virulence genes in comparison to the wild type. Deletion of *rpoS* (Δ*rpoS*) in *E coli* O104:H4 Δ*stx2* and typical EAEC resulted in a similar effect. Moreover, both *rpoS* ATG > ATA and Δ*rpoS* were found associated with stronger virulence-related phenotypes in comparison to the respective wild types. In particular, *rpoS* ATG > ATA caused enhanced epithelial barrier disruption, whereas Δ*rpoS* was characterized by enhanced bacterial cell aggregation, biofilm formation and host-pathogen interaction such as adherence and inflammation. We also demonstrated that the effect of RpoS on EAEC-specific virulence gene expression in *E. coli* O104:H4 is primarily mediated via repression of the major virulence activator AggR at the transcriptional level (Berger et al., 2024).

Given RpoS participation in diverse cellular processes and the strain-dependence of its functions, we wished to explore in this study the broader regulatory role of RpoS in *E. coli* O104:H4. We therefore performed gene set enrichment (GSE) analysis in combination with phenotype microarrays to analyse the impact of the *rpoS* ATG > ATA SNP on global gene expression. Furthermore, we included phenotypic investigations with a Δ*rpoS* mutant to confirm the role of RpoS on carbon source utilization.

## Materials and methods

### Bacterial strains

*E. coli* O104:H4 strain LB226692, isolated from a HUS patient during the 2011 outbreak in Germany (Mellmann et al., 2011), is referred to in this study as the wild type and is a progenitor of the strains described below. *E. coli* O104:H4 *rpoS* ATG > ATA had acquired a SNP in the start codon of *rpoS* during laboratory cultivation (preparation of a new glycerol stock from a random single colony) with no selection conditions applied (Berger et al., 2024). The *rpoS* deletion mutant *E. coli* O104:H4 Δ*stx2* Δ*rpoS* (Berger et al., 2024) was constructed by recombineering of *E. coli* O104:H4 Δ*stx2* (Peng et al., 2022).

### Preparation of protein samples and immunoblot

Protein sample preparation and immunoblot were performed as previously described (Berger et al., 2024). Briefly, overnight cultures were diluted 1:1000 and grown for 3 h (log) or for 19 h (overnight) at 37 °C, 180 rpm. Total protein samples were prepared by resuspending bacterial cell pellets in 1x Laemmli buffer (Bio-Rad) to an optical density (OD) OD_600_/μl of 0.01. The samples were separated by SDS-PAGE on any KD Mini-Protean TGX stain-free precast gels (Bio-Rad) and transferred to a PVDF (polyvinylidenfluoride) membrane using the Trans-Blot® TurboTM RTA Mini PVDF Transfer Kit (Bio-Rad). The membranes were blocked in 5% skimmed milk for 1 h at room temperature and incubated overnight with primary anti-RpoS monoclonal antibody (Biolegend; #663706) at 4 °C. After a 2 h incubation with an alkaline-phosphatase-conjugated anti-mouse IgG secondary antibody (JacksonImmunoResearch, #115–055-003), the signals were developed with NBT/BCIP substrate (Roche). The Western blots were scanned with a Chemidoc System (Bio-Rad) and the signal intensities of the bands of interest were quantified using the Lane and Bands Analysis Tool from Image Lab Software version 4.0.1 (Bio-Rad).

### Transcriptome analysis and comparison with previously published data

Details about bacterial growth, RNA preparation, RNA-seq experiment and raw data processing have been previously published (Berger et al., 2024). Briefly, overnight cultures were diluted to OD_600_ of 0.005 in LB, and cells were grown at 37 °C, 180 rpm to logarithmic growth phase (log, OD_600_ of 0.4–0.5) and transition to stationary phase (transition, OD_600_ of 3–3.4). The following reasons were considered when choosing the growth phases for analysis: (i) transition was chosen, since in LB, RpoS reaches its maximum concentration at the beginning of stationary phase (Lange and Hengge-Aronis, 1994); (ii) log was included, since there has been increasing evidence for the role of RpoS already at earlier growth stages (Corona-Izquierdo and Membrillo-Hernandez, 2002; Dong et al., 2008b; Dudin et al., 2013); (iii) both growth phases were previously analyzed in the typical EHEC strain *E. coli* O157:H7 EDL933 (Dong and Schellhorn, 2009). Total RNA was extracted with TRIzol Reagent (Thermo Fisher Scientific) and genomic DNA was removed by Turbo DNase (Thermo Fisher Scientific). rRNA depletion, RNA fragmentation, cDNA library construction and Illumina NextSeq 500 sequencing (single-reads; 75 bp read length) were done by vertis Biotechnology AG, Germany. Strand specific cDNA was prepared via adapter ligation to 3’OH ends of fragmented RNA. Illumina raw reads were mapped to *E. coli* O104:H4 strain 2011C − 3493 (GCF_000299455.1) as a reference genome using READemption 1.0.5 (Förstner et al., 2014) and segemehl 0.3.4 (Hoffmann et al., 2009). The transcriptome data are available at GEO Series accession number GSE243699. Here, differential gene expression analysis was performed with DESeq2 package 1.40.2 (Love et al., 2014) in R using gene wise quantification tables (quantification of the number of reads overlapping with the locations of the annotation genes) generated with READemption and as previously done (Berger et al., 2024) with the following modification: read counts for genes intersecting position 0 in the reference sequences and thus annotated as join of two fragments (O3K_RS26100, O3K_RS25615, and O3K_RS26550) were combined resulting in a total of 5,080 *E. coli* O104:H4 input genes with entries. DESeq2 expression data were obtained for 5,075/5,078 genes with nonzero total read count (Dataset 1 and 2). To allow for comparison of *E. coli* O104:H4 transcriptome data with previously published expression data from *E. coli* K-12 MG1655 (GCF_000005845.2) and *E. coli* O157:H7 EDL933 (GCF_000006665.1), orthologs between the three *E. coli* strains were extracted with OrthoFinder (Emms and Kelly, 2019).

### GSE analysis

GSE analysis was performed with clusterProfiler 4.8.3 (Wu et al., 2021) in R using all genes with available DESeq2 expression data (Dataset 1 and 2). Gene Ontology (GO) GSE was performed using the function gseGO and default parameters (min.gs.size = 10, max.gs.size = 500, pvalue.cutoff = 0.05, padjust.method = “BH”; Benjamini-Hochberg). Since *E. coli* O104:H4 is not among the supported organisms with available OrgDb object, we beforehand generated annotation data for GCF_000299455.1 using eggNOG-mapper 2.1.12 and default parameters (Cantalapiedra et al., 2021). The obtained data from the GO GSE analysis were visualized using the dotplot and cnetplot functions. Kyoto Encyclopedia of Genes and Genomes (KEGG) GSE was performed using the gseKEGG function with default parameters (see above) and the organism parameter set to “esl” corresponding to *E. coli* O104:H4 strain 2011C − 3493. The obtained data from the KEGG GSE analysis were visualized using the dotplot, cnetplot and pathview functions.

### BIOLOG phenotype microarrays

BIOLOG phenotype microarrays using the PM1 MicroPlate™ Carbon Utilization Assay were performed and analyzed as previously described (Berger et al., 2019). Briefly, bacterial glycerol stocks were streaked and re-streaked on Columbia Blood Agar (Oxoid) and cells were suspended in IF-0 inoculation fluid to OD_600_ = 0.17. The PM1 carbon source plates were set up and incubated following the manufacturer’s instruction. The growth kinetics measurements were performed in a TECAN Infinite F200 instrument by determining the OD_595_ every 15 min for 24 h. The obtained data were analyzed and visualized using the opm package v.1.3.77 for R (Vaas et al., 2013). After importing of kinetic raw data and metadata integration, descriptive curve parameters were estimated using the do_aggr function. The clustered results from the curve parameter A (maximum curve height) for different substrates were visualized using the function heat_map. A statistical analysis was performed with the opm_mcp method, which internally accounts for multiple comparisons, and using the curve parameters A or Area Under the Curve (AUC). The respiration curves over time were generated using the xy_plot function.

### Growth experiments in minimal medium supplemented with single carbon sources

The bacterial inoculums were prepared as described above for the BIOLOG phenotype microarrays with the following modification: the cell suspensions were adjusted to OD_600_ = 0.17 using double distilled water. For co-culture experiments, the cell suspensions of *E. coli* O104:H4 Δ*stx2* and Δ*stx2* Δ*rpoS* were mixed in a 1:1 ratio based on OD_600_, with the exact ratio (used for analysis) determined by counting colony forming units per ml (CFU/ml). 25 μl of the bacterial inoculums were added to 125 μl of M9 medium supplemented with 0.4% carbon source (0.33% final concentration) in a 96-well plate. The following carbon sources were used: D-galactose (Sigma, #G0750), D-ribose (Sigma, #R7500), L-arabinose (Roth, #5118.2), L-alanine (Sigma; #05129), L-glutamine (Sigma, #49419), L-aspartic acid (Sigma, #A8949). The growth kinetics measurements were recorded in TECAN Infinite F200 instrument as described above. The lag phase duration was calculated using a microbial lag phase calculator (Smug et al., 2024) and the tangent method with pre-processing of the growth curve (smooth) and default parameters. The bacterial cell numbers at 0 h and/or 24 h were determined by CFU/ml. In co-culture experiments, *E. coli* O104:H4 Δ*stx2* and Δ*stx2* Δ*rpoS* were distinguished from each other by plating each dilution on LB (all bacteria = 100%) and LB containing 25 μg/ml gentamicin plates (*E. coli* O104:H4 Δ*stx2* Δ*rpoS*). Statistical analysis and graphs depicting these data were done using R version 4.5.1 (R Core Team, 2021).

## Results

### Overview of differentially regulated genes in *E. coli* O104:H4 *rpoS* ATG > ATA

**Figure 1:**
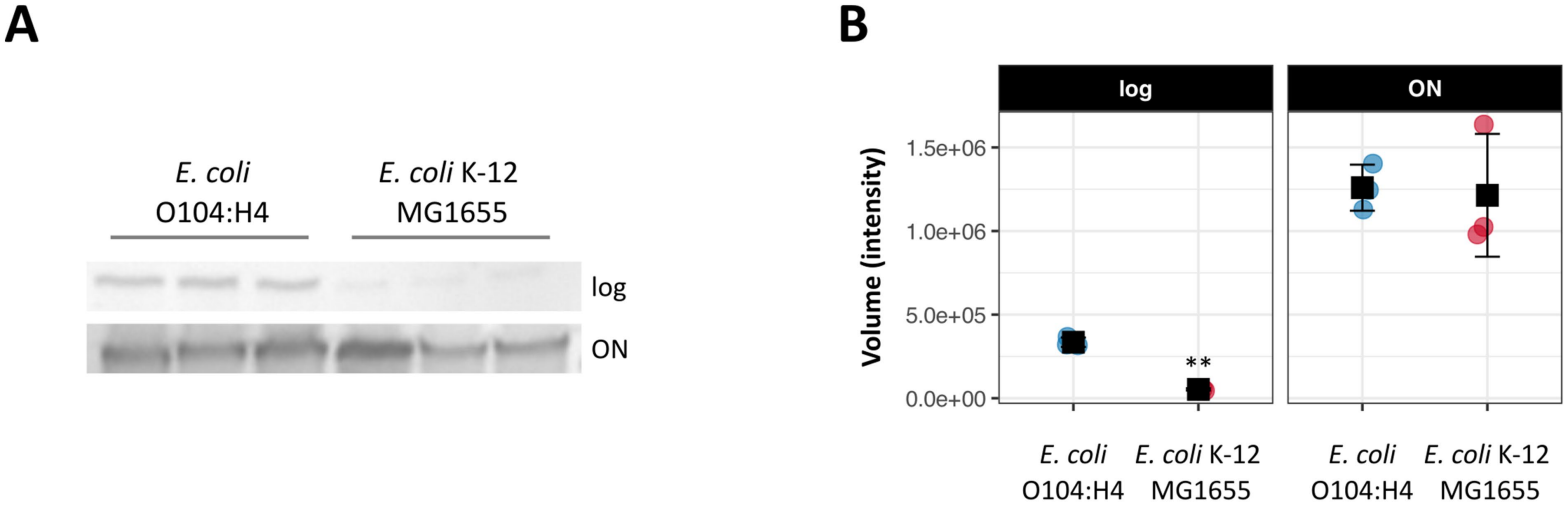
RpoS expression in *E. coli* O104:H4 and K-12 MG1655. **(A)** Expression of RpoS in log and in overnight (ON) cultures detected by semi-quantitative western blot analysis. **(B)** Quantification of RpoS expression. The immunoblots shown in **(A)** were quantified and Welch Two Sample *T*-test was used to assess the difference between samples (** *p* < 0.01). Graphs were created using R package ggplot2 (circles = three biological replicates per bacterial strain, squares = mean values, error bars = standard deviations) and final figures were created with Inkscape.

**Figure 2:**
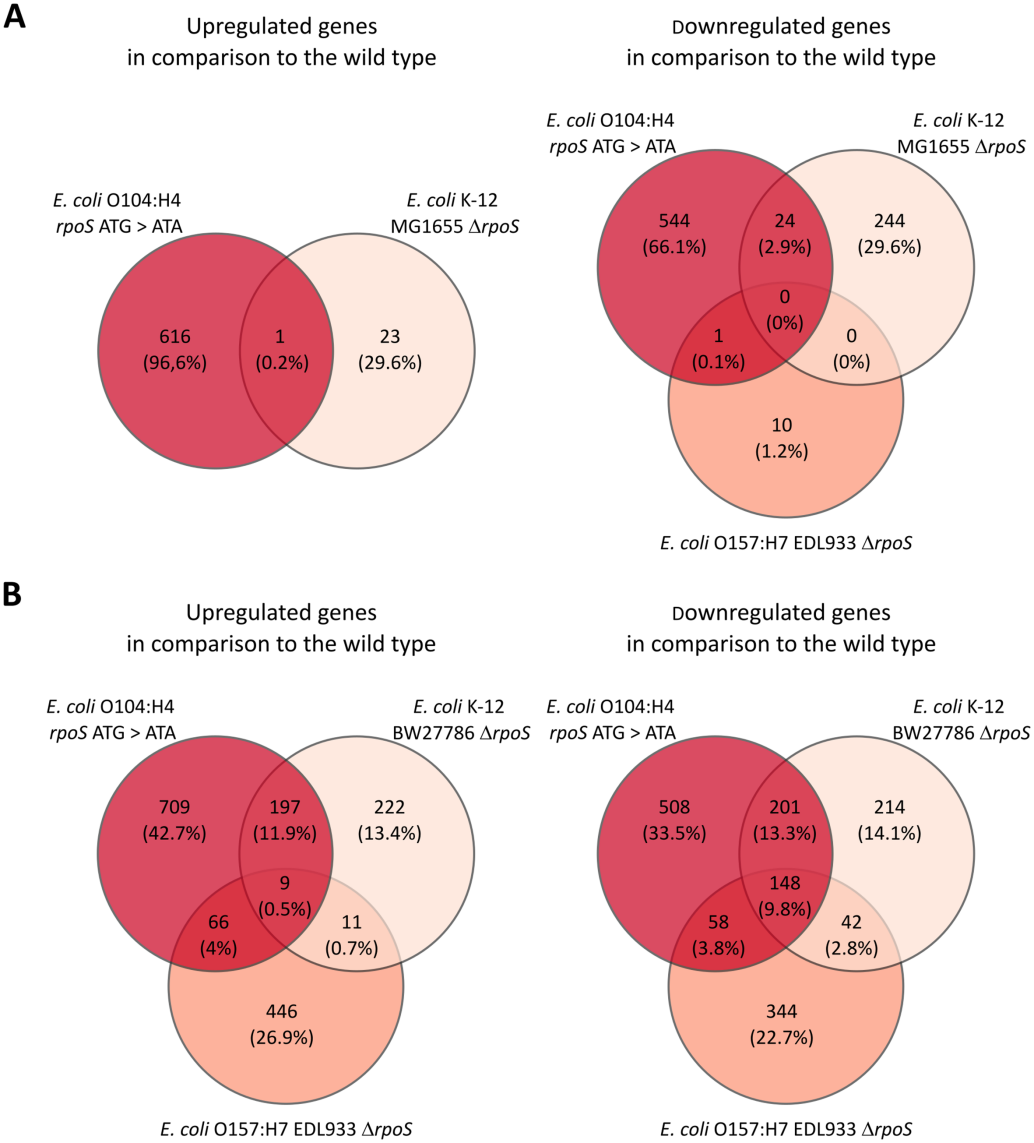
Overlap of RpoS regulated genes in *E. coli* O104:H4 and other *E. coli* strains. **(A)** Overlap of up- and downregulated genes in the *rpoS* mutant strains in comparison to the corresponding wild types during logarithmic growth. The Venn diagram shows the overlap between our data set and the one for *E. coli* K-12 MG1655 (Dong et al., 2008b) and *E. coli* O157:H7 EDL933 (Dong and Schellhorn, 2009). **(B)** Overlap of up- and downregulated genes in the *rpoS* mutant strains in comparison to the corresponding wild types in (transition to) stationary phase. The Venn diagram shows the overlap between our data set and the one for *E. coli* K-12 BW27786 (Wong et al., 2017) and *E. coli* O157:H7 EDL933 (Dong and Schellhorn, 2009).

We have recently subjected *E. coli* O104:H4 wild type and *rpoS* ATG > ATA cells grown to logarithmic growth phase (log; OD_600_ = 0.4–0.5) and to transition to stationary phase (transition; OD_600_ = 3.0–3.4) to quantitative RNA-seq (Berger et al., 2024). Using DESeq2 (Love et al., 2014), 1,186 (22%) of *E. coli* O104:H4 genes were found to be differentially expressed (|log2fold| > 1, padj < 0.01) in log, i.e., 617 up- and 569 downregulated genes in the *rpoS* ATG > ATA mutant strain (Dataset 1). Interestingly, the impact of *rpoS* ATG > ATA allele on *E. coli* O104:H4 gene expression during logarithmic growth appeared to be much stronger than the one described for Δ*rpoS* in *E. coli* K-12 MG1655 and EDL933, where 292 (6%) and 11 (<0.01%) of the annotated genes, respectively, were found to be differentially regulated in microarray analyses (Dong et al., 2008b; Dong and Schellhorn, 2009). Furthermore, semi-quantitative western blot revealed ~6-fold higher levels of RpoS in *E. coli* O104:H4 in comparison to MG1655 in this growth phase (Figure 1). There was only a small overlap between the RpoS-regulated genes in *E. coli* O104:H4 and MG1655 or EDL933 during log (Figure 2A; Dataset 1). Reflecting the increasing RpoS expression with progression of *E. coli* O104:H4 growth (Berger et al., 2024), we detected stronger impact of *rpoS* ATG > ATA on gene expression in transition, i.e., 1,896 genes (35%) were found to be differentially expressed with 981 being up- and 915 downregulated genes (Dataset 2). Comparison with previously published RNA-seq data of a derivative of the *E. coli* K-12 strain BD792 in late stationary phase, where the expression of 1,044 genes (23%) was reported to be RpoS-regulated (Wong et al., 2017), revealed that 206 up- and 349 downregulated genes were found shared in both *rpoS* mutant backgrounds (Figure 2B; Dataset 2). Notably, less similarity was detected between our data and the microarray data from EDL933 in stationary phase, where 1,124 RpoS-regulated genes were previously reported (20%; Dong and Schellhorn, 2009). Namely, 75 up- and 206 downregulated common genes were found in *E. coli* O104:H4 *rpoS* ATG > ATA and EDL933 Δ*rpoS* in comparison to the respective wild type strains (Figure 2B; Dataset 2). Lastly by comparing the list of differentially expressed genes in log and transition in *E. coli* O104:H4 *rpoS* ATA > ATG, we found that the majority of differentially regulated genes were growth phase specific and only 339 (27%) up- and 190 (15%) downregulated genes were shared in both growth phases, among them being numerous upregulated virulence genes (Supplementary Figure 1; Berger et al., 2024).

### Impact of *rpoS* ATG > ATA allele on global gene expression in *E. coli* O104:H4 in log

**Figure 3:**
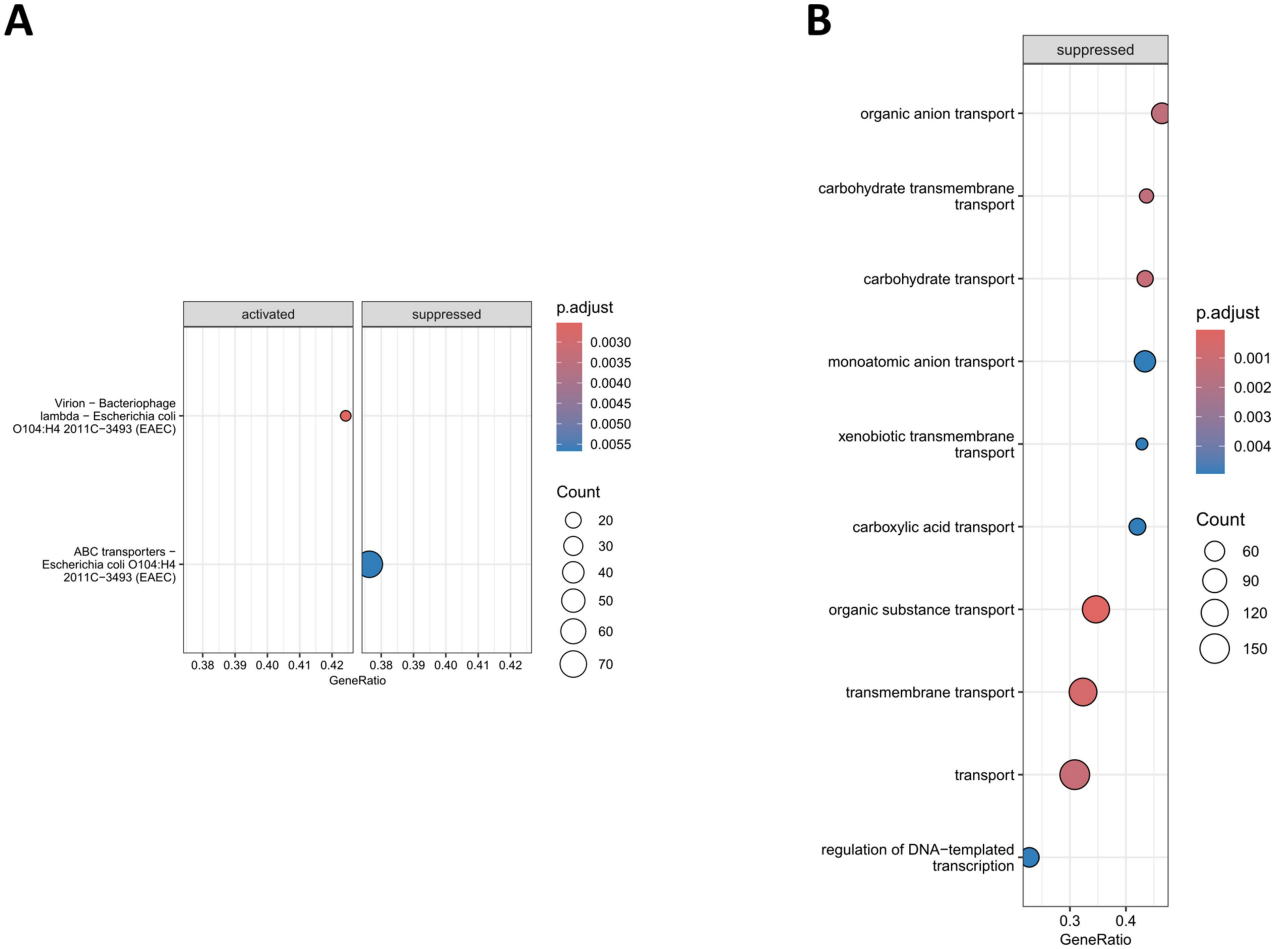
Dot plot of enriched set of genes in *E. coli* O104:H4 *rpoS* ATG > ATA in comparison to the wild type during log. **(A)** Enriched KEGG pathways. **(B)** Enriched GO terms. Top 10 enriched GO terms are shown. The *x*-axis shows the enrichment ratio (GeneRatio) and the dots are colored and sized based on the given adjusted *p*-value (*p*.adjust) and gene count legend, respectively.

Next, we performed a GSE analysis using the R package clusterProfiler (Wu et al., 2021) in order to learn more about the impact of *rpoS* ATG > ATA on global gene expression in *E. coli* O104:H4. A significant enrichment in only two KEGG pathways was detected in log (Figure 3A). The “Virion—bacteriophage lambda” pathway, with genes belonging to prophages C, F and H (Ahmed et al., 2012), was found to be activated in the mutant strain, while genes coding for “ABC transporters” were suppressed with the monosaccharide transporters of ribose (*rbsDACB*) and *sn*-glycerol 3-phosphate (*ugpBAE*) showing the strongest difference (Supplementary Figures 2, 3A; Dataset 3). The GO GSE revealed only suppressed pathways in *E. coli* O104:H4 *rpoS* ATG > ATA, with 9 out of the 10 most significant GO terms being also transport-related ones, e.g., “Organic anion transport,” “Carbohydrate transport,” “Transmembrane transport” (Figure 3B). The acquisition of the *rpoS* ATG > ATA SNP resulted in the downregulation of genes coding for transporters of a variety of substrates. For example, among the most strongly downregulated transport-related genes were the ones involved in the uptake of fucose (*fucP*), sorbitol (*srlA*), nucleosides (*nupG*), thiamine (*thiP*), C4-dicarboxylates, i.e., fumarate, succinate, L-aspartate, L-malate (*dctA*) and L-serine (*sdaC*; Supplementary Figure 4; Dataset 4). Several of the transport-related genes were also previously detected to be downregulated in MG1655 Δ*rpoS* in comparison to the wild type strain, e.g., *fucP*, *rbsC*, *malK* (uptake of maltose; Dong et al., 2008b). In addition, the GO GSE analysis revealed that genes belonging to “Regulation of DNA-templated transcription” were significantly repressed in *E. coli* O104:H4 *rpoS* ATG > ATA (Figure 3B; Supplementary Figure 4; Dataset 4). Notably, the majority of repressed genes coding for transcription regulators were involved in carbon source uptake and metabolism. Interestingly, approximately 40% of the transport and transcription regulation-related repressed genes from the GO GSE analysis were previously reported to be activated by the global regulator cyclic AMP receptor protein, CRP (Salgado et al., 2024), which gene was also found to be downregulated in the *rpoS* ATG > ATA mutant. Thus, our analysis revealed an RpoS-dependent activation of carbon uptake-related transport and regulator genes in *E. coli* O104:H4 during log.

### Impact of *rpoS* ATG > ATA allele on global gene expression in *E. coli* O104:H4 in transition

**Figure 4:**
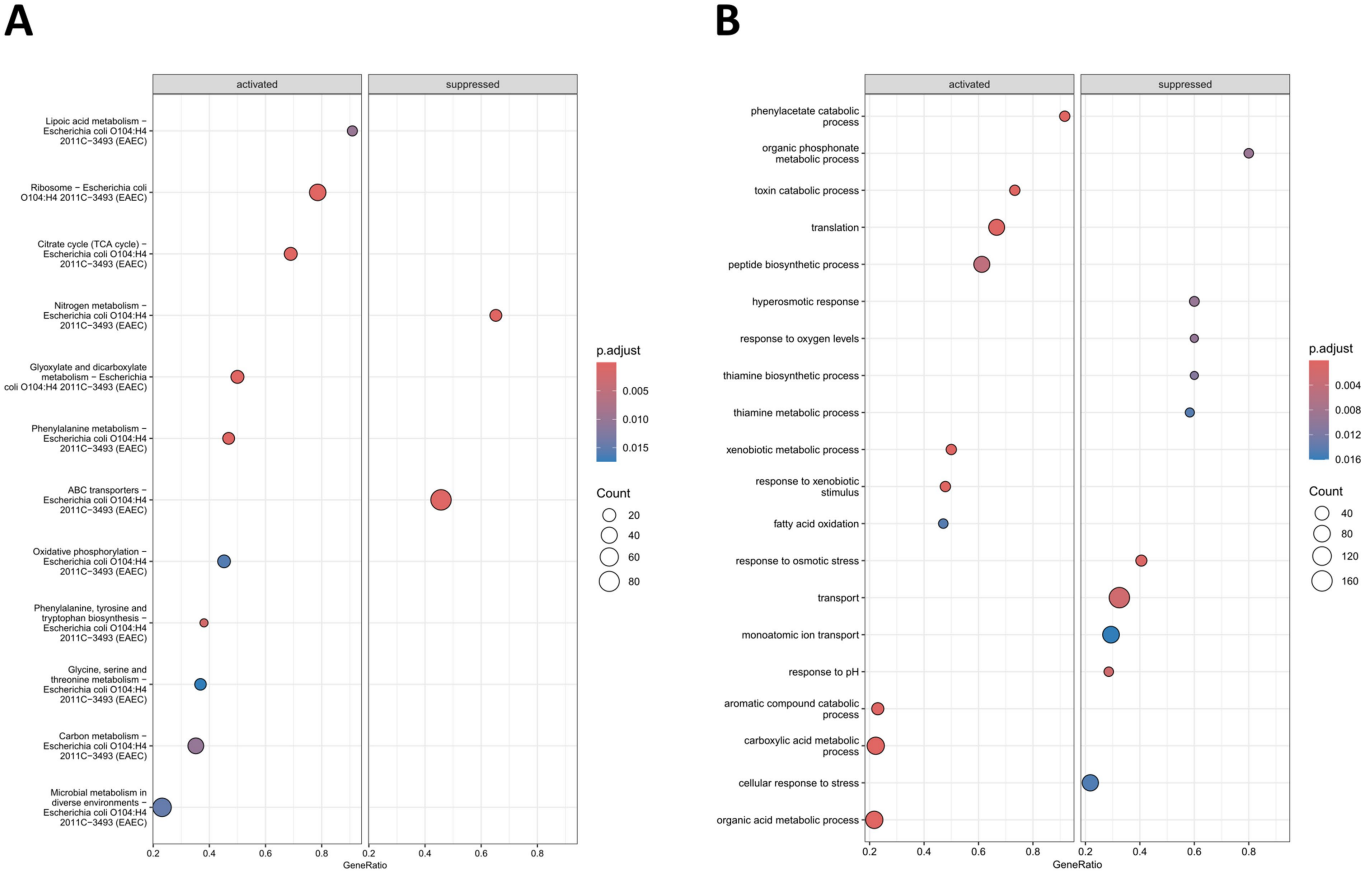
Dot plot of enriched set of genes in *E. coli* O104:H4 *rpoS* ATG > ATA in comparison to the wild type during transition. **(A)** Enriched KEGG pathways. **(B)** Enriched GO terms. Top 10 enriched KEGG pathways and GO terms are shown. The *x*-axis shows the enrichment ratio (GeneRatio) and the dots are colored and sized based on the given adjusted *p*-value (*p.adjust*) and gene count legend, respectively.

We detected much stronger enrichment of both KEGG pathways and GO terms in transition (Figure 4), which was expected due to the higher number of regulated genes by RpoS at this growth phase in comparison to log. Interestingly, the presence of the *rpoS* ATG > ATA allele led to the activation of genes belonging mainly to metabolism-related KEGG pathways (9 out of the 10 most significant pathways) in *E. coli* O104:H4 in transition (Figure 4A). The genes with the strongest activation in the *rpoS* ATG > ATA mutant were part of the carbohydrate metabolism pathway “Glyoxylate and dicarboxylate metabolism” (Supplementary Figure 5A; Dataset 5). Genes of three amino acid metabolic KEGG pathways, i.e., “Phenylalanine metabolism” (Supplementary Figure 5B), “Phenylalanine, tyrosine and tryptophan biosynthesis” (Supplementary Figure 5C) and “Glycine, serine and threonine metabolism” (Supplementary Figure 5D) were also found to be overall upregulated. Moreover, we detected activation of genes encoding “TCA cycle” (Supplementary Figure 5E) and “Oxidative phosphorylation” enzymes (Supplementary Figure 5F), e.g., fumarase A (*fumA*), succinyl-CoA synthetase (*sucABCD*), succinate dehydrogenase (*sdhCDAB*) and cytochrome bo complex (*cyoABCDE*; Dataset 5). Lastly, all genes listed to be part of the “Lipoic acid metabolism” showed upregulation (Supplementary Figure 5G; Dataset 5). Apart from metabolism-related KEGG pathways, the *rpoS* ATG > ATA mutation was also accompanied by activation in the translation pathway “Ribosome” (Supplementary Figure 5H; Dataset 5), with genes encoding components of both the 50S and 30S subunit of the ribosome found to be upregulated. Only two KEGG pathways were found repressed in *E. coli* O104:H4 *rpoS* ATG > ATA in comparison to the wild type (Figure 4A). Similar to our observations in log and previously described for EDL933 Δ*rpoS* in stationary phase (Dong and Schellhorn, 2009), genes of “ABC transporters” were found overall repressed by *rpoS* ATG > ATA in transition (Supplementary Figure 3B; Dataset 5). Nevertheless, a closer look revealed the opposite expression pattern of several transport genes during early and late growth stages, e.g., *rbsDAB* were now found upregulated in the *rpoS* ATG > ATA strain in transition. Lastly, genes belonging to the “Nitrogen metabolism” pathway were downregulated in the *rpoS* ATG > ATA mutant (Supplementary Figure 5I; Dataset 5).

The GO GSE analysis confirmed the activation of “translation” and numerous metabolism-related GO terms and the repression of transport-related ones (Figure 4B; Supplementary Figure 6; Dataset 6). In addition, the analysis revealed the downregulation of genes belonging to the “organic phosphonate metabolic process” and “thiamin biosynthetic/metabolic process.” The rest of the suppressed GO terms (5 out of the 10 most significant ones) detected in the *rpoS* ATG > ATA mutant in transition were stress-related, which was expected since RpoS is well known to control the general stress response in gram-negative bacteria (Hengge, 2010). The most populated stress-induced GO term was “cellular response to stress,” which also shared genes with the other enriched stress-related pathways, i.e., “response to osmotic stress” and its sub-class “hyperosmotic response,” “response to oxygen levels” and “response to pH” (Supplementary Figure 6; Dataset 6). Therefore, our data indicated that the negative regulation of metabolic pathways and the positive regulation of transport- and stress-related ones were the hallmarks of RpoS effects on global gene expression in *E. coli* O104:H4 in transition.

### Impact of *rpoS* ATG > ATA on carbon source utilization of *E. coli* O104:H4

**Figure 5:**
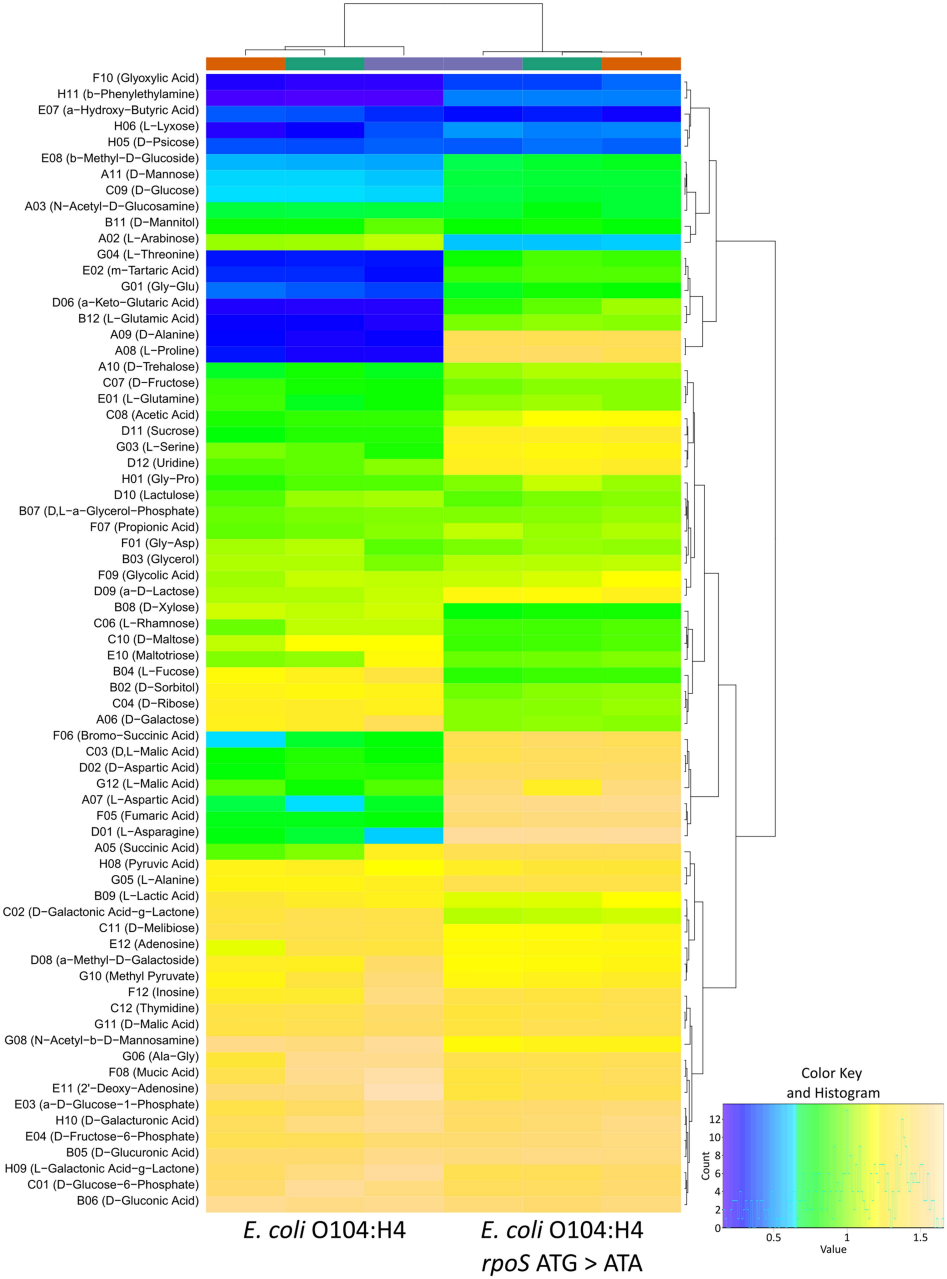
Heatmap of the respiration potential of *E. coli* O104:H4 wild type and *rpoS* ATG > ATA analyzed with the BIOLOG PM1 assay. Only substrates, which were assimilated at least by one of the strains are shown. Three biological replicates per strain were analyzed. The provided color key shows the degree of respiration (based on A; maximum curve height). The tree on top of the map shows the relationship between the analyzed samples and the tree on the side shows the relationship between the substrates.

Since the majority of RpoS-regulated KEGG pathways and GO terms in our gene set enrichment analysis were carbon source transport- or metabolism-related, we next addressed the question if the above-described changes in the transcriptome could lead to significant differences in the carbon source utilization. For this purpose, we subjected *E. coli* O104:H4 wild type and *rpoS* ATG > ATA to BIOLOG phenotype microarray PM1 assay. The strains showed no respiration with 24 of the 95 tested substrates and differentially utilized 43 of them. The acquisition of the *rpoS* ATG > ATA SNP resulted in the reduced respiration with 14 and increased respiration with 29 substrates (Figure 5; Supplementary Table 1). Interestingly, the majority of the substrates, with which the *rpoS* ATG > ATA strain showed reduced respiration were sugars (eight) or sugar derivatives (three). Among them were sugars known to be present in mucus (Doranga et al., 2024), i.e., L-arabinose, L-fucose, D-galactose, D-ribose and D-maltose. Nevertheless, the mutant assimilated five other sugars more efficiently than the wild type, e.g., sucrose and D-trehalose. There were numerous consistencies between our RNA-seq data and these sugar-related phenotypes. For example, the reduced respiration of the *rpoS* ATG > ATA mutant with L-arabinose was in agreement with the downregulation of the *araBDA* catabolism operon and the regulatory gene *araC* in log, while its increased respiration with sucrose was supported by the downregulation in log of the repressor gene *cscR* (O3K_RS07595) and the upregulation in transition of *cscK* (O3K_RS07600) encoding a fructokinase and *cscB* (O3K_RS07505) coding for a non-PTS sucrose-H^+^ symporter.

The carbon sources utilized more efficiently by the *rpoS* ATG > ATA mutant than the wild type in the BIOLOG PM1 analysis fell into three categories: amino acids (10), dipeptides (two), and organic acids (nine, Figure 5; Supplementary Table 1). The acquisition of the *rpoS* ATG > ATA SNP led to a remarkable increase in the respiration properties with amino acids, i.e., the mutant utilized virtually all amino acids included in the PM1 panel more efficiently (10 out of the 12 amino acids; with two amino acids both strains showed no respiration). Interestingly, the *rpoS* ATG > ATA mutant could also efficiently assimilate amino acids with which the wild type showed no detectable respiration, e.g., L − proline, D − alanine (Supplementary Figure 7). Furthermore, the mutant utilized more efficiently the carboxylic acids acetic acid and glyoxylic acid and seven C4-dicarboxylic acids, e.g., fumaric acid and bromo-succinic acid (Figure 5; Supplementary Table 1). These phenotypes were in agreement with our RNA seq data and in particular with the activation of pathways like the TCA cycle, amino acid and organic acid metabolic pathways at transition (Figure 4). Taken together, the acquisition of the *rpoS* ATG > ATA SNP in *E. coli* O104:H4 was accompanied by a shift in the carbon source utilization/metabolic respiration fingerprint of the strain, i.e., from stronger respiration with sugars/derivatives (intact *rpoS*) to stronger respiration with amino acids and organic acids (*rpoS* ATG > ATA). These observations suggested that RpoS positively regulates the assimilation of certain sugars and sugar derivatives, e.g., constituents of the mucus, and negatively regulates the assimilation of amino acids and organic acids in *E. coli* O104:H4.

### Impact of Δ*rpoS* on carbon source utilization of *E. coli* O104:H4

**Figure 6:**
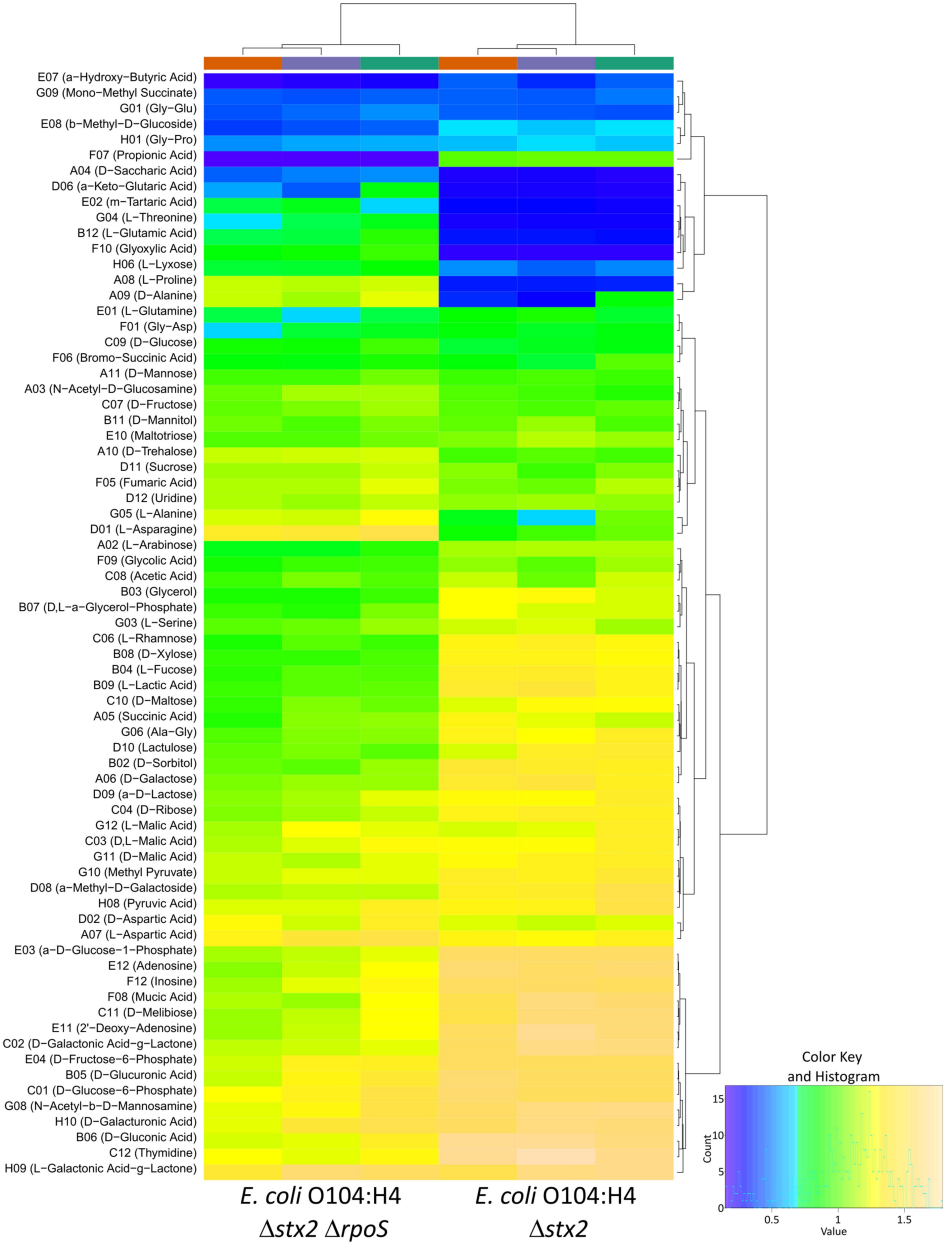
Heatmap of the respiration potential of *E. coli* O104:H4 Δ*stx2* and Δ*stx2* Δ*rpoS* ATG > ATA strains analyzed with the BIOLOG PM1 assay. Only substrates, which were assimilated at least by one of the strains are shown. Three biological replicates per strain were analyzed. The provided color key shows the degree of respiration (based on A; maximum curve height). The tree on top of the map shows the relationship between the analyzed samples and the tree on the side shows the relationship between the substrates.

Since *rpoS* ATG > ATA is a knockdown mutation (Berger et al., 2024), we next wished to analyze the effect of *rpoS* deletion on carbon source utilization of *E. coli* O104:H4 using the BIOLOG PM1 assay. Both *E. coli* O104:H4 Δ*stx2* and Δ*stx2* Δ*rpoS* showed no respiration with 24 substrates, whereas they differentially assimilated 45 substrates (Figure 6; Supplementary Table 2). The Δ*rpoS* mutant displayed reduced respiration with 33 and stronger one with 12 substrates in comparison to the wild type. As expected, the positive effect of RpoS on the utilization of sugar (10) and sugar derivatives (14) was now much more pronounced than what we had observed when comparing *E. coli* O104:H4 wild type and *rpoS* ATG > ATA (compare Figure 6 with Figure 5). Notably, the Δ*rpoS* mutant showed in addition reduced respiration with acids of sugars also known to be present in mucus (Doranga et al., 2024), i.e., D-gluconic acid, D-glucuronic acid and D-galacturonic acid. Furthermore, the deletion of *rpoS* resulted in the significantly stronger utilization of four of the five tested nucleosides, e.g., 2′ − deoxy−adenosine and thymidine (Figure 6; Supplementary Table 2).

In contrast, the negative effect of RpoS on the utilization of amino acids and organic acids was less pronounced when comparing *E. coli* O104:H4 Δ*stx2* and Δ*stx2* Δ*rpoS*. Namely, the deletion of *rpoS* resulted in more efficient respiration with only six of the 10 amino acids and three of the nine organic acids previously detected to be utilized more efficiently upon the acquisition of the *rpoS* ATG > ATA SNP (compare Figure 6 with Figure 5). A closer look at the metabolic kinetics revealed in the case of the missing C4-dicarboxylic amino acids L- and D-aspartic acid and all C4-dicarboxylic acids (succinic, malic, fumaric and bromo−succinic acid) stronger respiration of the Δ*rpoS* strain at early growth stages, while the wild type was able to catch up only at the end of the 24 h BIOLOG assay (Supplementary Figure 7). Indeed, a statistical analysis using the area under the curve revealed overall stronger respiration of *E. coli* O104:H4 Δ*stx2* Δ*rpoS* with these C4-dicarboxylates (Supplementary Table 2), which are known to be oxidized in the TCA cycle during aerobic growth (Unden and Kleefeld, 2004). Moreover, a comparison of the metabolic fingerprint of all analyzed strains revealed that the two *rpoS* mutant strains, i.e., *E. coli* O104:H4 *rpoS* ATG > ATA and *E. coli* O104:H4 Δ*stx2* Δ*rpoS*, and the two *rpoS* wild type strains, i.e., *E. coli* O104:H4 and *E. coli* O104:H4 Δ*stx2*, clustered together (Supplementary Figure 8). Taken together, the impact of Δ*rpoS* on carbon source utilization of *E. coli* O104:H4 appeared to be similar but not the same as the one of the *rpoS* ATG > ATA allele. In particular, the analysis of *E. coli* O104:H4 Δ*stx2* and Δ*stx2* Δ*rpoS* indicated an even stronger positive effect of RpoS on the assimilation of sugars and sugar derivatives and revealed a new one on nucleosides, while confirmed RpoS negative regulation of amino acids and suggested rather a transient negative effect on the assimilation of C4-dicarboxylates.

### Impact of Δ*rpoS* on single carbon source growth of *E. coli* O104:H4

**Figure 7:**
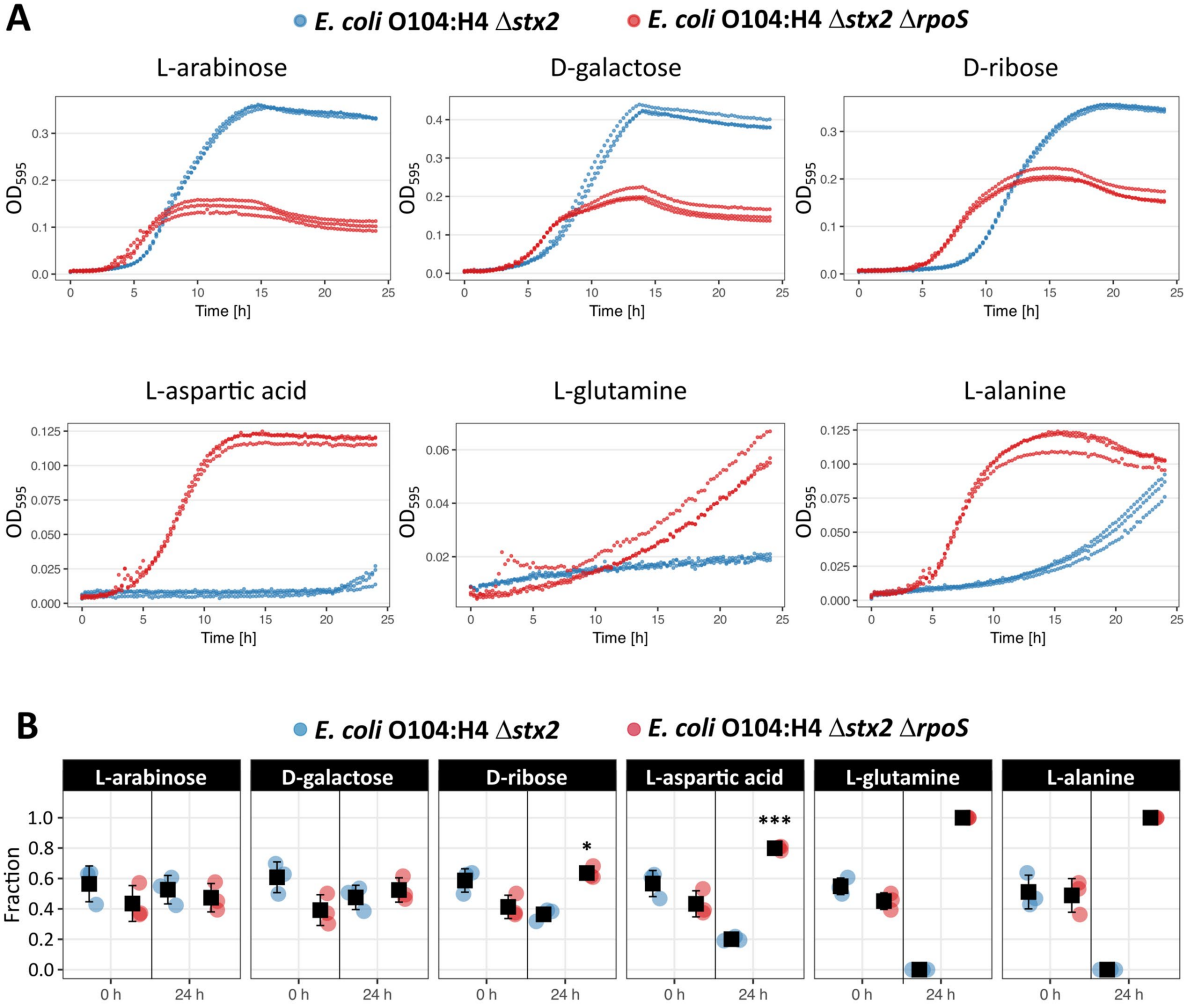
Growth experiments with *E. coli* O104:H4 Δ*stx2* and Δ*stx2* Δ*rpoS* in M9 medium supplemented with a single carbon source. **(A)** Growth kinetics of single culture experiments. The experiments were done with three biological replicate per strain and the OD_595_ values were plotted over time. The final colony forming units per ml (CFU/ml) counts after 24 h of incubation are shown in Supplementary Figure 9A. **(B)** Fraction of *E. coli* O104:H4 Δ*stx2* and Δ*stx2* Δ*rpoS* during co-culture experiments. The experiments were done with three co-cultures of different biological replicates of the strains. The fraction was calculated at the beginning (0 h) and end (24 h) of the experiment based on CFU/ml counts on LB (both strains) and LB with gentamicin plates (*E. coli* O104:H4 Δ*stx2* Δ*rpoS*). Paired *T*-test was used to assess the difference between samples. Graphs were created using R package ggplot2 (circles = three biological replicates per bacterial strain, squares = mean values, error bars = standard deviations; *p < 0.05, **p < 0.01, ***p < 0.001) and final figures were created with Inkscape. The CFU/ml counts are shown in Supplementary Figure 9B.

We last addressed the question whether the effect of RpoS on carbon source utilization also has an impact on the growth of *E. coli* O104:H4. For this purpose, we cultured *E. coli* O104:H4 Δ*stx2* and Δ*stx2* Δ*rpoS* alone or together in a 1:1 ratio in minimal medium supplemented with single carbon sources, i.e., sugars and amino acids as examples for the positive and negative role of RpoS on their utilization, respectively. In single culture experiments, the *E. coli* O104:H4 Δ*stx2* exhibited better growth, as judged by higher maximum OD and final CFU/ml, when cultured with the sugars D-galactose, D-arabinose and D-ribose, whereas the Δ*rpoS* mutant grew better with the amino acids L-aspartic acid, L-glutamine and L-alanine when comparing the two strains (Figure 7A; Supplementary Figure 9A). These carbon source-specific growth phenotypes were in agreement with the utilization efficiencies seen previously in our BIOLOG PM1 analysis, except for L-Glutamine, where only a positive effect of its assimilation was detected with the *rpoS* ATG > ATA allele, but not Δ*rpoS* (Figures 5 and 6; Supplementary Figure 7). Interestingly, the growth curves of the Δ*rpoS* mutant in minimal medium supplemented with sugars were characterized by a shorter lag phase (Supplementary Table 3).

The expected effect of the *rpoS* deletion on the growth in co-culture was detected only with amino acids as a sole carbon source. The competitive advantage of *E. coli* O104:H4 Δ*stx2* Δ*rpoS* with L-Alanine and L-Glutamine was even so strong that we failed to detect the wild type in the co-culture after 24 h. On the contrary, *E. coli* O104:H4 Δ*stx2* and Δ*stx2* Δ*rpoS* were found both at the beginning and the end of the experiment to be in approx. 1:1 ratio when grown with D-galactose and D-arabinose, whereas the Δ*rpoS* mutant was even characterized by a slight competitive advantage in the presence of D-ribose (Figure 7B; Supplementary Figure 9B). These results suggested that the deletion of *rpoS* provides both growth and competitive advantage in the presence of amino acids as a sole carbon source in the *E. coli* O104:H4 Δ*stx2* background.

## Discussion

In this study, we aimed to analyze the impact of RpoS on global gene expression in the highly pathogenic *E. coli* O104:H4. Transcriptomic analysis revealed that RpoS is vastly involved in the regulation of metabolic and carbon utilization-related genes. Consistently, phenotypic experiments confirmed its central role in carbon source assimilation, growth and competition.

The expression of several factors reported to modulate RpoS levels and activity (Bouillet et al., 2024), was affected by the acquisition of the *rpoS* ATG > ATA SNP in *E. coli* O104:H4 (Dataset 1 and 2). Specifically, the gene encoding the atypical response regulator RssB that mediates RpoS protein stability by promoting its degradation (Pratt and Silhavy, 1996) was downregulated in *E. coli* O104:H4 *rpoS* ATG > ATA during transition. Furthermore, genes coding for Ira anti-adaptors functioning as inhibitors of RssB activity (Bougdour et al., 2008) were upregulated; *iraM* during log and transition and *iraP* during transition. This differential expression pattern might contribute to RpoS stability and accumulation in the *rpoS* ATG > ATA mutant, thereby partially explaining how a non-canonical ATA start codon, which provides only up to 1% of the translation efficiency compared with ATG (Hecht et al., 2017), could result in just a ≤ 5-fold reduction in RpoS protein levels (Berger et al., 2024). Furthermore, the gene encoding Crl—an RNAP holoenzyme assembly factor promoting the association of RpoS with the RNAP core (Banta et al., 2013)—was upregulated during both growth phases and might serve as an adaptation mechanism in *E. coli* O104:H4 *rpoS* ATG > ATA to cope with the reduced RpoS levels.

In this study, we found only a small overlap between the RpoS regulons in *E. coli* O104:H4, K-12 and EDL933 (Figure 2), which is in agreement with the highly versatile, strain-specific role of RpoS in gene expression that was previously proposed by others (Schellhorn, 2020). The positive regulation of stress-related pathways by RpoS, however, appears to be a conserved feature in these and other gram-negative bacteria (Figure 4; Bouillet et al., 2024). Interestingly, *E. coli* O104:H4 shared more RpoS-regulated genes with the K-12 strains than with the typical EHEC strain EDL933. For example, genes belonging to central metabolic pathways like the TCA cycle and oxidative phosphorylation, were found to be repressed by RpoS in both *E. coli* O104:H4 and K-12, but not regulated in EDL933. Furthermore, amino acid utilization genes were described to be activated by RpoS in EDL933, whereas found repressed by RpoS in both *E. coli* O104:H4 and K-12 (Dong and Schellhorn, 2009; Wong et al., 2017). Whether this is due to the higher sequence similarity of the *E. coli* O104:H4 *rpoS* allele to that of K-12 (299/300 identical amino acids) compared to the one of EDL933 (298/300), or due to other characteristics of the specific genetic background, remains to be further investigated. Notably, the effect of RpoS on global gene expression in typical EAEC, with which *E. coli* O104:H4 share higher genome sequence similarities than with typical EHEC (Brzuszkiewicz et al., 2011; Mellmann et al., 2011), has not been yet subjected to analysis. In this context, it would be interesting to address the question to what extent Stx2 phage carriage and toxin production in both typical and hybrid EHEC could contribute to or be modulated by, respectively, strain-specific regulatory effects of RpoS. Namely, both RpoS and Stx phage carriage have the capacity to reprogram the bacterial central metabolism and carbon source utilization (Berger et al., 2019; Schellhorn, 2020), while the SOS response, which is required for Stx phage induction and production, and RpoS regulons are functionally linked (Dapa et al., 2017).

The higher fraction of RpoS regulated genes in *E. coli* O1O4:H4 during logarithmic growth (23%) in comparison to the one described for MG1655 (6%) (Dong et al., 2008b) and EDL933 (<0.01%) (Dong and Schellhorn, 2009) is striking. Differences in the experimental set up, e.g., the methodologies (microarrays instead of RNA-seq) used in these studies may contribute to this effect to some extent. Nevertheless, we also detected a significantly stronger RpoS expression in *E. coli* O104:H4 than in MG16655 in log but not in overnight cultures (Figure 1). Higher RpoS levels in comparison to MG1655 have been previously reported also for EDL933 in logarithmically growing cells, however this appears to have only a minor impact on gene expression in this typical EHEC strain (Dong and Schellhorn, 2009). Therefore, the higher number of RpoS-dependent genes in *E. coli* O1O4: H4 during log might indicate a previously unrecognized strong effect of RpoS on gene expression in pathogenic strains already at early growth stages. It is of course possible, that the exceptionally large number of RpoS-regulated genes in *E. coli* O104:H4 is resulting from indirect effects, as suggested by the enrichment of genes coding for known transcription regulators in this growth phase (Figure 3B). Moreover, we cannot exclude the possibility that *E. coli* O104:H4 encodes a so far unidentified RpoS-dependent transcriptional regulator that is absent in MG1655 and EDL933.

During transition, the majority of activated KEGG pathways and many of the GO terms associated with the acquisition of the *rpoS* ATG > ATA SNP were found to be metabolism-related (Figure 4; Supplementary Figures 5, 6). The extensive regulation of metabolic genes by RpoS at this growth phase has been reported in both *E. coli* K-12 and Salmonella (Levi-Meyrueis et al., 2014; Wong et al., 2017) and it is considered to be a central aspect of the bacterial general stress response (Bouillet et al., 2024). Importantly, our data indicates that RpoS negatively regulates the expression of genes belonging to central metabolic pathways like the TCA cycle and oxidative phosphorylation, as well as to amino acid biosynthesis and degradation pathways, lipoic acid, and glyoxylate and dicarboxylate metabolism in *E. coli* O104:H4 (Figure 4A; Supplementary Figure 5). Two main mechanisms have been proposed to mediate RpoS-dependent negative regulation: competition with the primary sigma factor RpoD for the core polymerase (Farewell et al., 1998) or competition with RpoD for stationary phase promoter (Cho et al., 2014). The downregulation of genes encoding aerobic metabolism meets two major demands of stationary phase cells: (i) decreased demand for energy metabolism due to lower availability of nutrients/growth arrest; and (ii) increased demand for protection against accumulating reactive oxygen species, natural but harmful by-products of aerobic metabolism (Chang et al., 2002; Patten et al., 2004). In addition to that, the negative regulation of the TCA cycle by RpoS enables *rpoS* mutant cells to scavenge nutrients that feed into these pathways more efficiently (Schellhorn, 2020).

Transport-related genes are the only class activated by RpoS in *E. coli* O104:H4 during both analyzed growth phases, although with some growth-specific differences in individual genes (Figures 3 and 4; Supplementary Figure 3; Dataset 3–6). The RpoS-dependent activation of genes involved in carbon transport can promote not only stress survival, with glutamate/gamma-aminobutyrate uptake/export and putrescine and arginine uptake being important in acid and osmotic stress responses, respectively, but also growth on non-optimal carbon sources in bacteria (Bouillet et al., 2024). Indeed, we detected that RpoS activates the expression of numerous transport systems for sugars, which can be utilized by the cell as alternatives to glycose (Martinez-Gomez et al., 2012; Aidelberg et al., 2014), in *E. coli* O104:H4 during log (Supplementary Figures 3, 4). Moreover, the majority of genes encoding transcription factors, including CRP, the main mediator of catabolite control in *E. coli* (Gosset et al., 2004), that were activated by RpoS during log are also involved in regulating carbon uptake and/or metabolism (Supplementary Figure 4; Dataset 4). These observations further highlight the importance of RpoS acting as a metabolic switch in *E. coli* O104:H4.

Our phenotypic experiments confirmed a profound impact of RpoS on carbon utilization-related phenotypes, i.e., metabolic respiration, growth and competition in *E. coli* O104:H4 (Figures 5–7). BIOLOG PM1 experiments with both *rpoS* ATG > ATA and ∆*rpoS* strains revealed a weaker respiration of the mutants with sugars, whereas an enhanced one above all with amino acids in comparison to the respective wild types. As expected, the positive effect of RpoS on sugar assimilation was more pronounced upon complete absence of the alternative sigma factor. However, the *rpoS* ATG > ATA allele (reduced RpoS) was associated with stronger phenotypes than ∆*rpoS* (no RpoS) in the presence of amino and carboxylic acids—TCA cycle substrates—when compared to the wild type (Figures 5, 6; Supplementary Tables 1, 2; Supplementary Figure 7). This might indicate that the negative effect of RpoS on their assimilation might be a transient one, i.e., weakening/disappearing after the transition to stationary phase, as it seems to be the case for C4 amino acids and dicarboxylic acids (Supplementary Figure 7). Furthermore, genes involved in their assimilation might respond with different sensitivity to varying levels of RpoS amounts (Wong et al., 2017). It has to be noted here that the experiments evaluating the impact of ∆*rpoS* were done in a ∆*stx2* background. Presence of Stx2 might eventually have an additional, indirect impact on the metabolism of *E. coli* O104:H4, since the toxin can inhibit also bacterial ribosomes and translation (Skinner and Jackson, 1998; Suh et al., 1998). Nevertheless, the expression of *stx2* is strictly dependent on phage activation (Tyler et al., 2004), i.e., the toxin is produced by cells, which will promptly lyse, and such an indirect effect on metabolism is therefore expected to have limited biological significance. Moreover, we currently cannot exclude the possibility that apart from resulting in reduction in RpoS amounts, the *rpoS* ATG > ATA allele might also alter binding properties of RpoS to promoters (Gowrishankar et al., 2003; Berger et al., 2024). Nevertheless, ∆*rpoS* conferred a clear growth and competitive advantage over the wild type in M9 medium supplemented with amino acids, including L-Glutamine, with which no significant difference was seen in the BIOLOG PM1 analysis (Figures 5, 6; Supplementary Tables 1, 2; Supplementary Figure 7). This suggests that the nutrient environment might have a strong impact on RpoS-dependent phenotypes in *E. coli* O104:H4, as previously described for the *E. coli* K-12 strain W3110 (Farrell and Finkel, 2003). Notably, the growth advantage of the wild type when incubated alone is not reflected by a fitness advantage in competition against the ∆*rpoS* strain in M9 supplemented with sugars after 24 h of incubation. It appears very likely that the shorter lag phase of the ∆*rpoS* mutant could compensate for its growth defect and allow the mutant to reach similar cell numbers as the wild type in co-culture experiments (Figure 7; Supplementary Figure 8; Supplementary Table 3). Another possibility is that metabolic, or enzymatic properties of the wild type are assisting the mutant to overcome its growth defect, e.g., by facilitating the uptake of limiting nutrients in a shared environment.

RpoS is central for cell survival in *E. coli* (Lange and Hengge-Aronis, 1991), nevertheless, some *rpoS* mutants exhibit a growth advantage in stationary phase (GASP) and become dominant in *in vitro* mixed cultures with the wild type (Zambrano et al., 1993). Notably, *rpoS* and other mutations enhancing the catabolism of amino acids, which are a primary source of nutrients derived from dead bacteria in stationary cultures, confer this GASP phenotype (Zinser and Kolter, 1999). Indeed, our analysis indicates that *rpoS* mutations in *E. coli* O104:H4 enable the strain to both assimilate and compete for amino acids more efficiently *in vitro* (Figures 5–7). It is very likely that this has been the reason for the acquisition of the *rpoS* ATG > ATA SNP in *E. coli* O104:H4 during laboratory cultivation (Berger et al., 2024). Interestingly, human *E. coli* O157:H7 isolates are characterized by enrichment of mutations in *rpoS* when compared to bovine and food isolates, suggesting that conditions within the human host may favor the selection of *rpoS* mutants. The bovine gastrointestinal tract and soil/manure/food might require functional wild type *rpoS* allele for survival in these more hostile conditions. Conditions in the human gut, on the other hand, might favor the acquisition of *rpoS* mutations, which could result in the more efficient nutrient scavenging of less abundant nutrients (van Hoek et al., 2013). This is supported by the hypothesis that a main driving force for acquisitions of *rpoS* mutations is a trade-off between high stress resistance (functional *rpoS*) and more efficient nutrient scavenging (mutated *rpoS*) (Ferenci, 2003; King et al., 2004).

RpoS modulates the colonization properties of both commensal and pathogenic *E. coli* (Krogfelt et al., 2000; Price et al., 2000; Barron et al., 2020), most likely as a result of its role in both stress resistance and nutrient utilization. Indeed, *E. coli* encounters various stressors during the passage through the gastrointestinal tract, particularly the acidic environment of the stomach, while its ability to colonize the intestine depends on the efficiency with which a given strain utilizes available carbon sources (Conway and Cohen, 2015). Our data suggests that *rpoS* mutants of *E. coli* O104:H4 might be compromised in their ability to survive the passage through the stomach (Figure 4B), similarly to what was proposed for EDL 933 (Price et al., 2000). In the gut, *E. coli* feeds primarily on simple monosaccharides and disaccharides derived from dietary fibers and the glycoprotein mucin, the major component of the mucus layer (Doranga et al., 2024). Presence of a functional *rpoS* allele in *E. coli* O104:H4 is associated with significantly increased utilization of sugars including some commonly found in the mucus (Figures 5 and 6; Supplementary Tables 1, 2; Supplementary Figure 7), however failed to confer a competitive advantage over the *rpoS* deletion mutant in co-culture experiments (Figure 7), and thus it remains unclear if it might be crucial for efficient colonization of the strain *in vivo*. On the contrary, acquisition of *rpoS* mutations in *E. coli* O104:H4 might ensure better competition for amino acids (Figure 7), which are also mucin constituents and sources for both carbon and nitrogen in the gut (Doranga et al., 2024). Amino acid catabolism does not play a major role in the colonization of commensal *E. coli* (Chang et al., 2004). Yet, efficient assimilation of dietary amino acids like L-serine might provide fitness and growth advantage for pathogenic strains against commensal competitors in the inflamed gut (Kitamoto et al., 2020). Moreover, the acquisition of *rpoS* mutations might be beneficial for *E. coli* O104:H4 in the gut via the increased expression of EAEC-specific virulence factors (Berger et al., 2024), among them the serine protease with mucinolytic activity Pic, which has been shown to contribute to intestinal colonization and enhance growth in mucus (Harrington et al., 2009). However, the exact impact of *rpoS* mutations on the ability of *E. coli* O104:H4 to colonize the gut remains to be investigated.

Taken together, our results define a profound role of RpoS on gene expression and carbon source utilization in *E. coli* O104:H4, further highlighting its role as a global regulator in pathogenic bacteria.

## Data Availability

The datasets presented in this study can be found in online repositories. The names of the repository/repositories and accession number(s) can be found in the article/Supplementary material.
