# Supplementary material for: RpoS impacts global gene expression and carbon source utilization in Escherichia coli O104:H4
Source: Front Microbiol. 2026 Jan 20;16:1758449. doi: 10.3389/fmicb.2025.1758449 (PMC12864459; doi:10.3389/fmicb.2025.1758449)
Supplement: Supplementary file 7 [file Supplementary_file_1.docx]

Supplementary Material

# Supplementary Data

**Dataset 1. Differentially regulated genes in *E. coli* O104:H4 wild type and *rpoS* ATG > ATA in log**

**Dataset 2. Differentially regulated genes in *E. coli* O104:H4 wild type and *rpoS* ATG > ATA in transition**

**Dataset 3. Genes belonging to enriched KEGG pathways in log**

**Dataset 4. Genes belonging to enriched GO terms in log**

**Dataset 5. Genes belonging to enriched KEGG pathways in transition**

**Dataset 4. Genes belonging to enriched GO terms in transition**

# Supplementary Figures and Tables

## Supplementary Figures

**
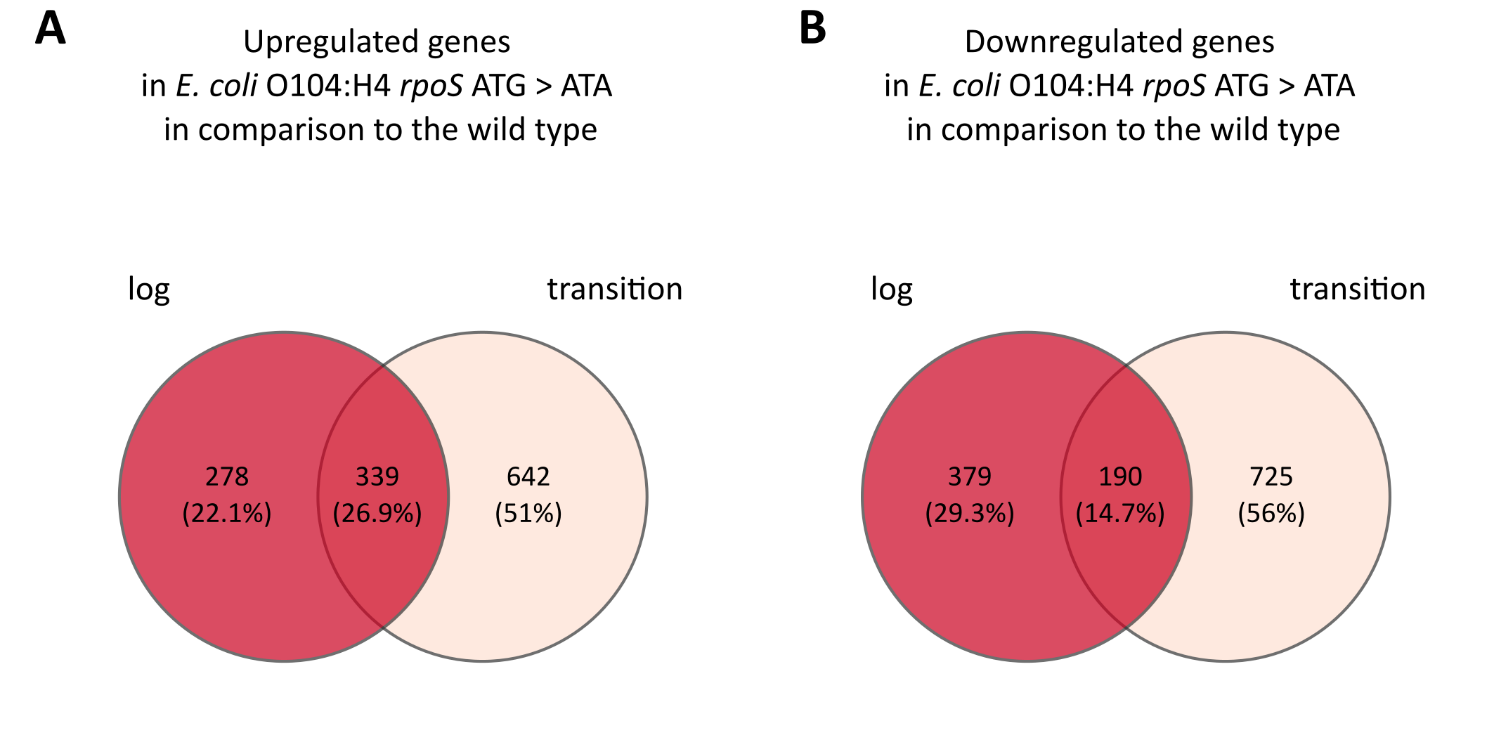
Supplementary Figure 1. Overlap of RpoS regulated genes in *E. coli* O104:H4 during log and transition. (A)** Overlap of genes found upregulated in *E. coli* O104:H4 *rpoS* ATG > ATA in comparison to the wild type. **(B)** Overlap of genes found downregulated in *E. coli* O104:H4 *rpoS* ATG > ATA in comparison to wild type.


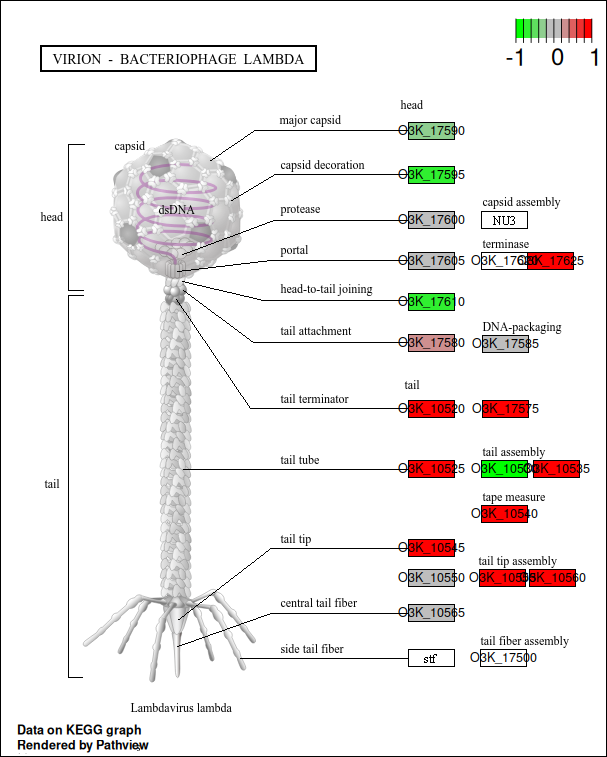


**Supplementary Figure 2. The KEGG pathway “Virion – bacteriophage lambda” found activated** **in *E. coli* O104:H4 *rpoS* ATG > ATA in comparison to the wild type during log.** Colored genes are present in the strain. Gene expression values were mapped to the given gradient color scale.


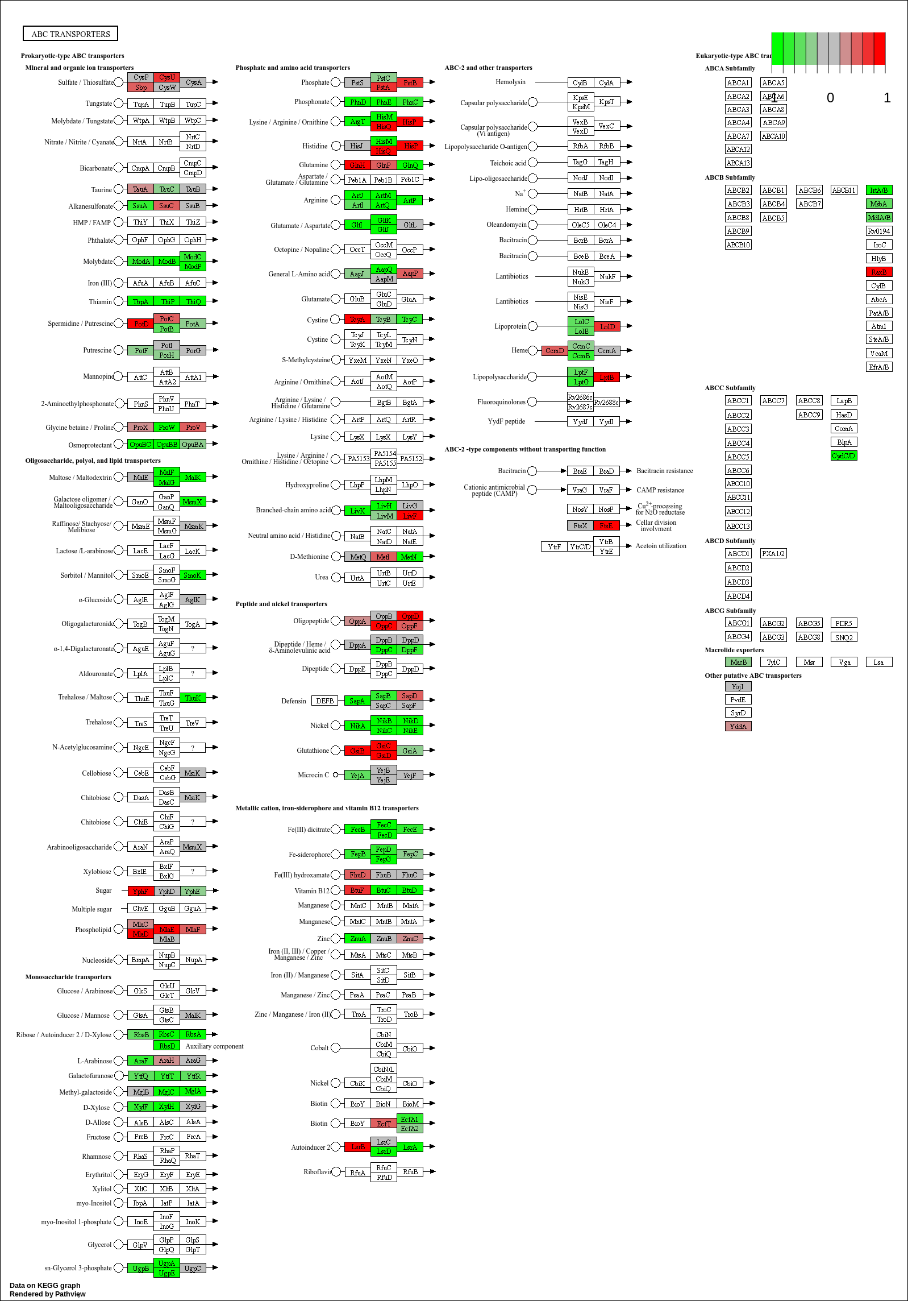

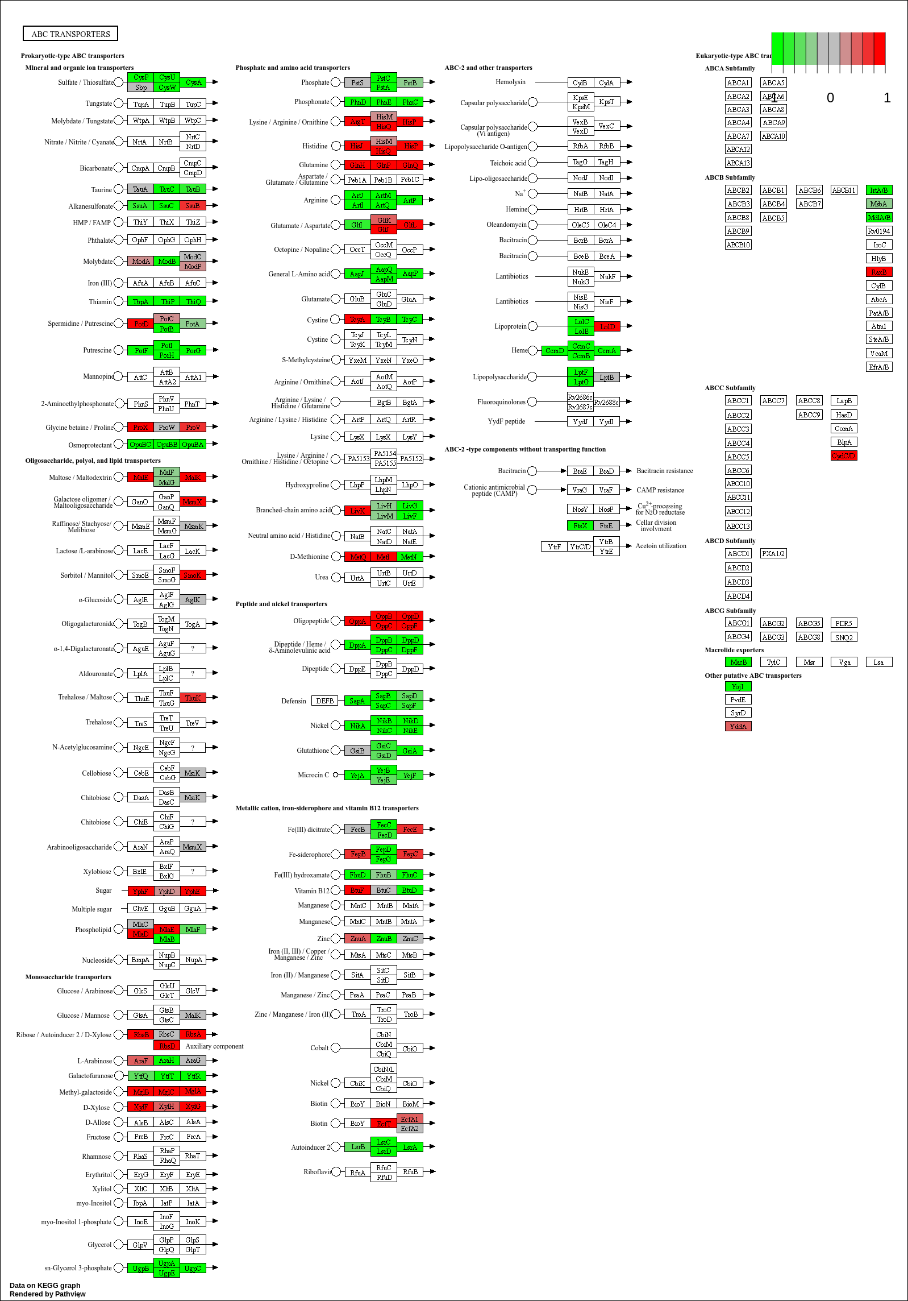
**Supplementary Figure 3. The KEGG pathway “ABC transporters” found suppressed in *E. coli* O104:H4 *rpoS* ATG > ATA in comparison to the wild type during log (A) and transition (B).** Genes for colored proteins are present in the strain. Gene expression values were mapped to the given gradient color scale.

A

B

**
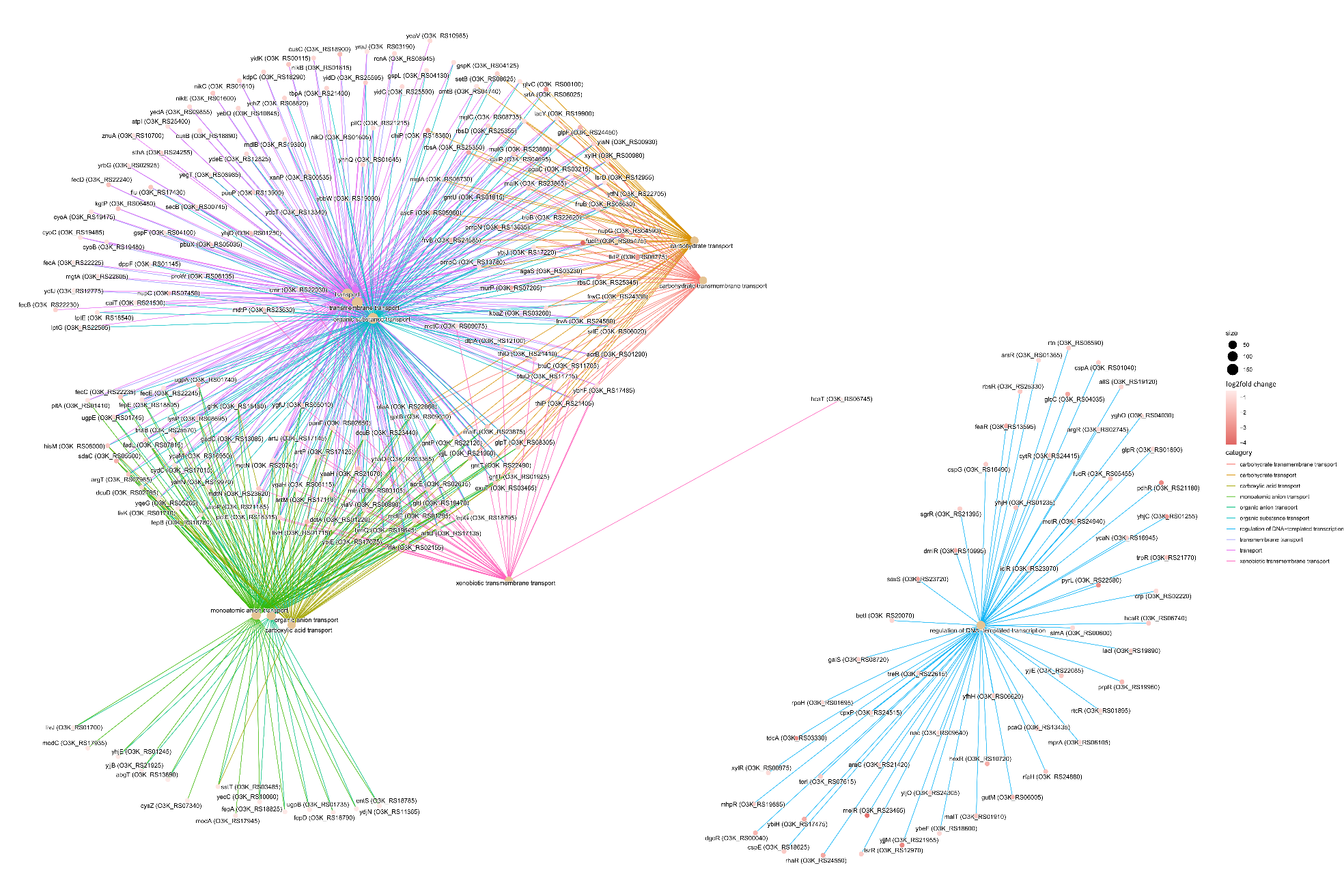
Supplementary Figure 4. Cnet plot of enriched GO terms in *E. coli* O104:H4 *rpoS* ATG > ATA in comparison to the wild type in log.**


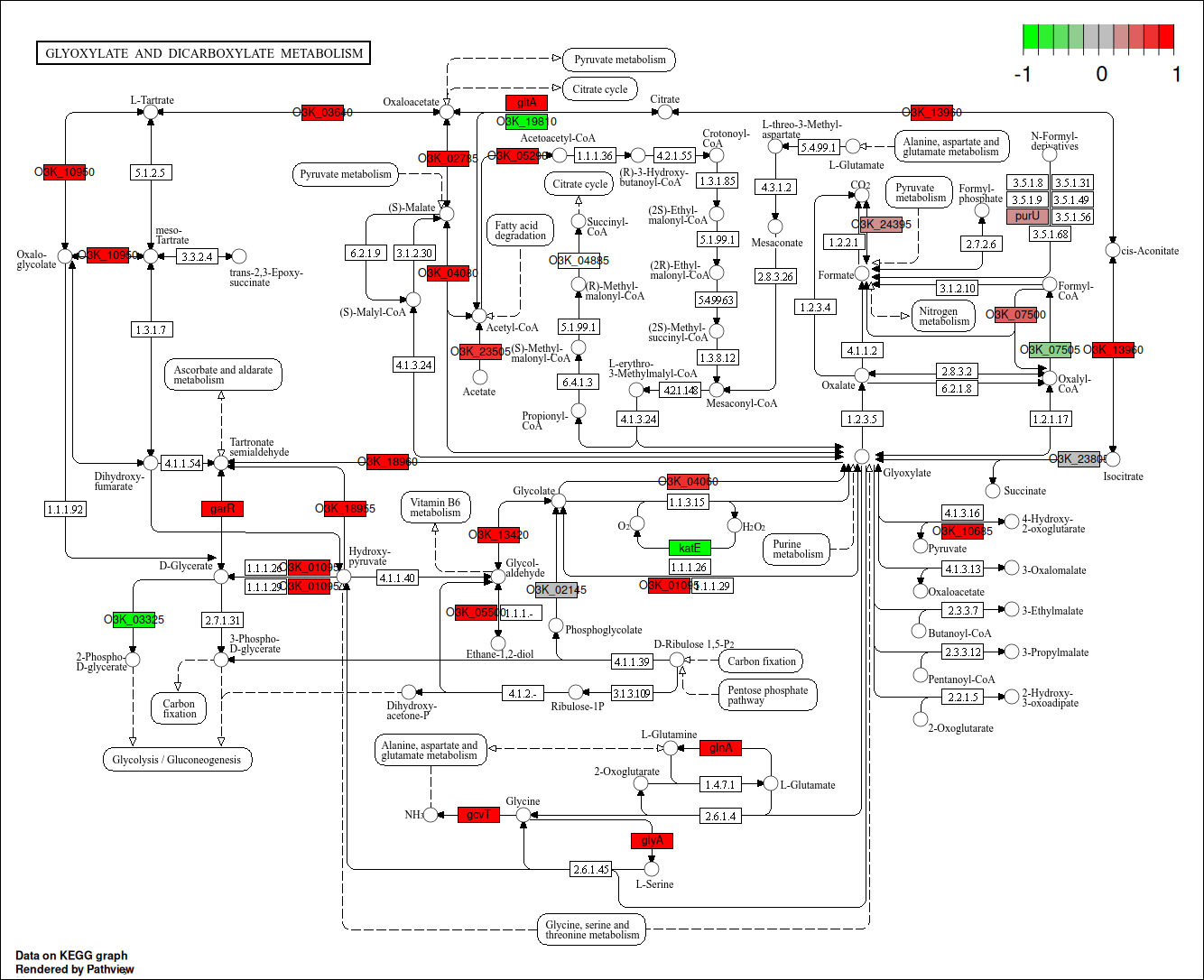


**Supplementary Figure 5. Enriched KEGG pathways in *E. coli* O104:H4 *rpoS* ATG > ATA in comparison to the wild type during transition. (A)** The KEGG pathway “Glyoxylate and dicarboxylate metabolism” found activated in *E. coli* O104:H4 *rpoS* ATG >ATA in comparison to the wild type during transition. Colored genes are present in the strain. Gene expression values were mapped to the given gradient color scale.


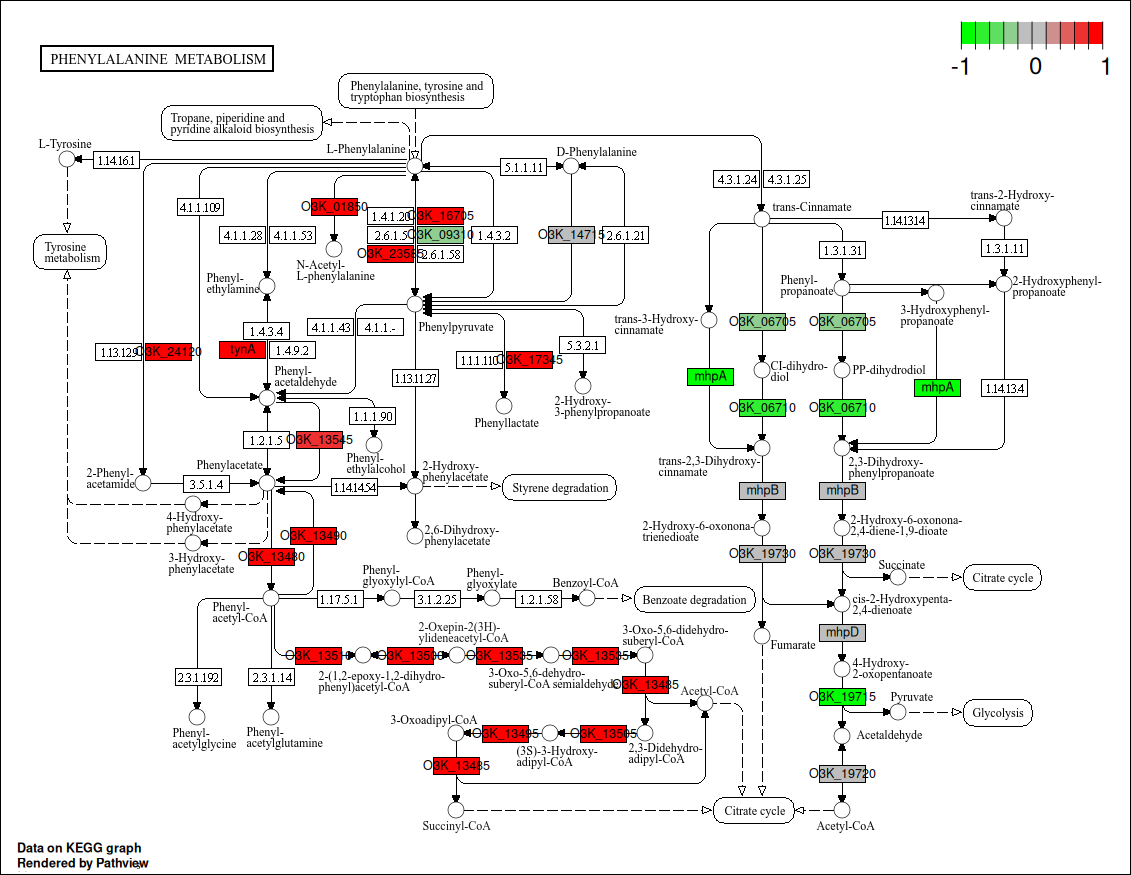


**(B)** The KEGG pathway “Phenylalanine metabolism” found activated in *E. coli* O104:H4 *rpoS* ATG > ATA in comparison to the wild type during transition. Colored genes are present in the strain. Gene expression values were mapped to the given gradient color scale.


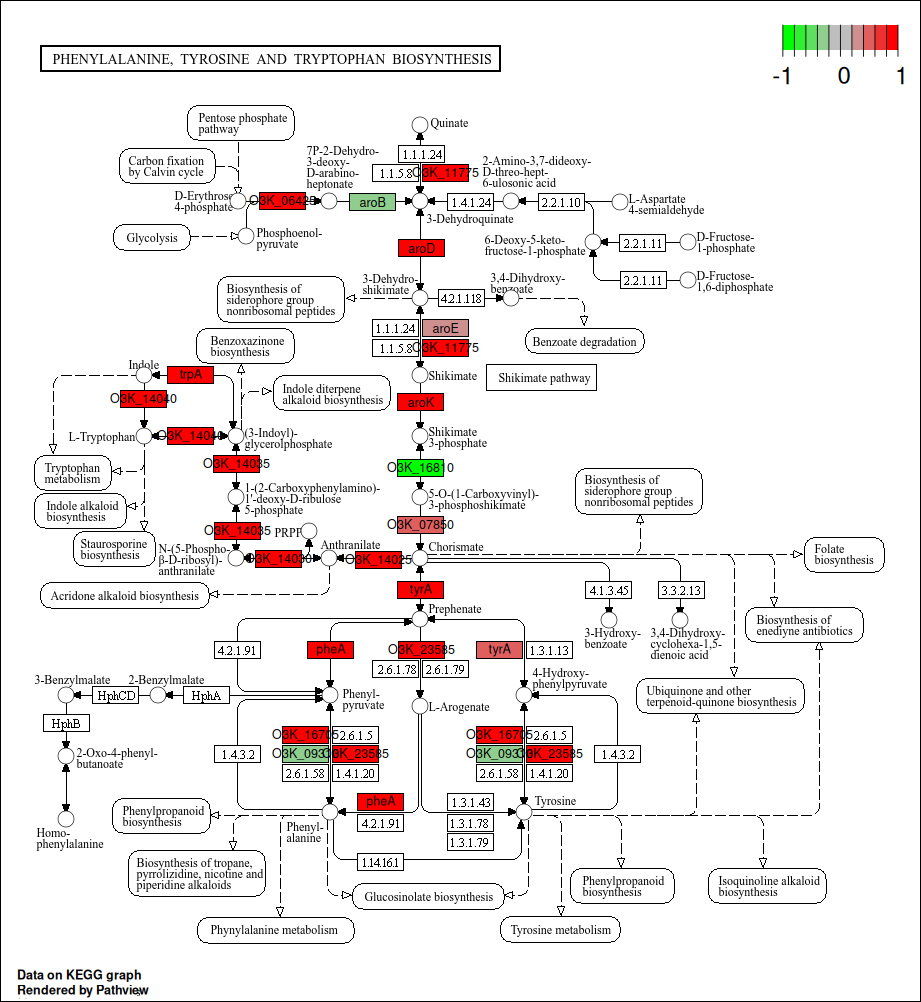


**(C)** The KEGG pathway “Phenylalanine, tyrosine and tryptophan biosynthesis” found activated in *E. coli* O104:H4 *rpoS* ATG > ATA in comparison to the wild type during transition. Colored genes are present in the strain. Gene expression values were mapped to the given gradient color scale.


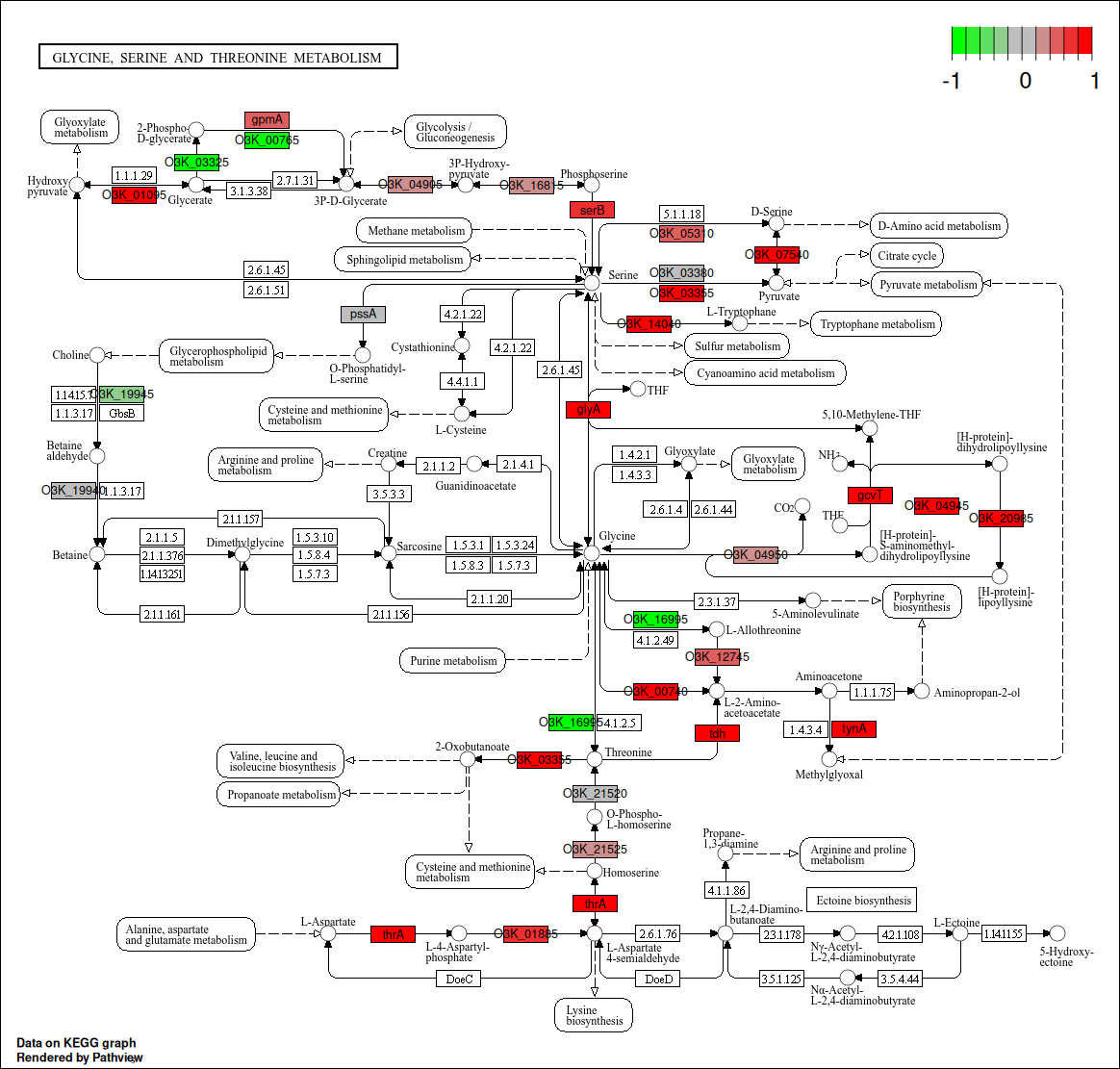


**(D)** The KEGG pathway “Glycine, serine and threonine metabolism” found activated in *E. coli* O104:H4 *rpoS* ATG > ATA in comparison to the wild type during transition. Colored genes are present in the strain. Gene expression values were mapped to the given gradient color scale.


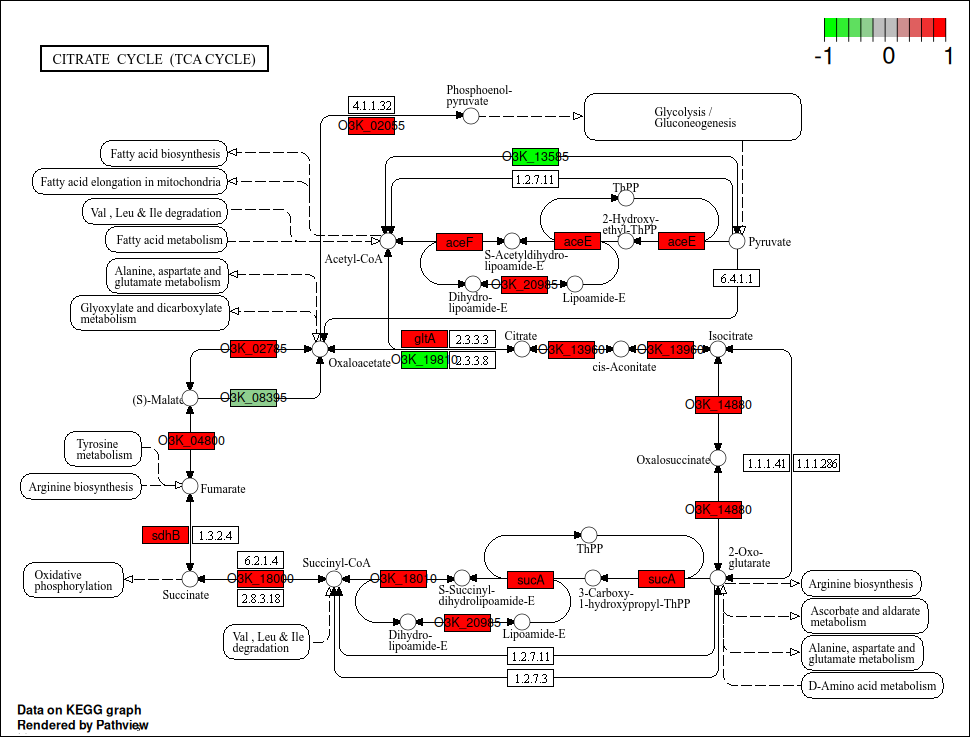


**(E)** The KEGG pathway “TCA cycle” found activated in *E. coli* O104:H4 *rpoS* ATG > ATA in comparison to the wild type during transition. Colored genes are present in the strain. Gene expression values were mapped to the given gradient color scale.


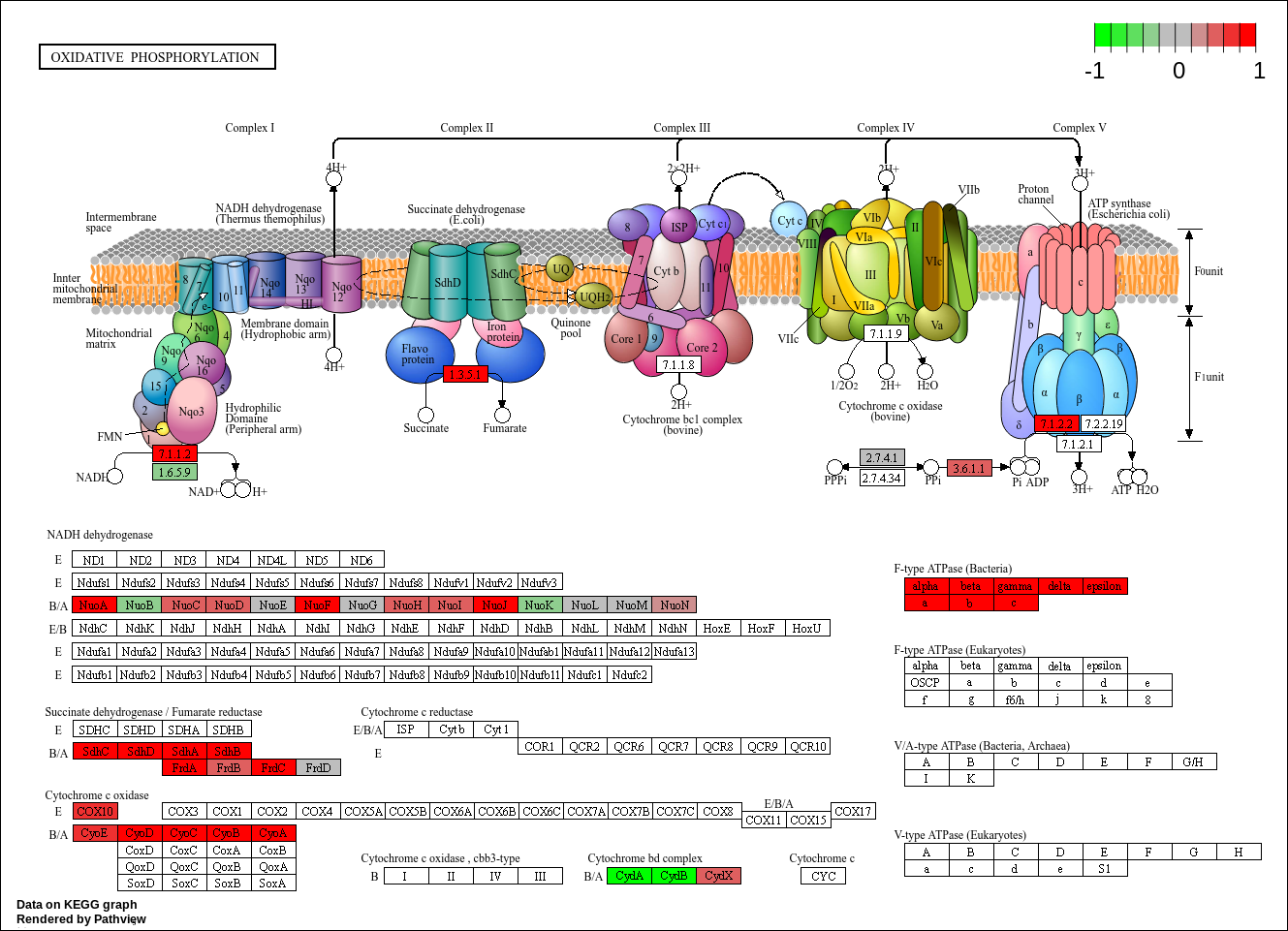


**(F)** The KEGG pathway “Oxidative phosphorylation” found activated in *E. coli* O104:H4 *rpoS* ATG > ATA in comparison to the wild type during transition. Colored proteins are present in the strain. Gene expression values were mapped to the given gradient color scale.


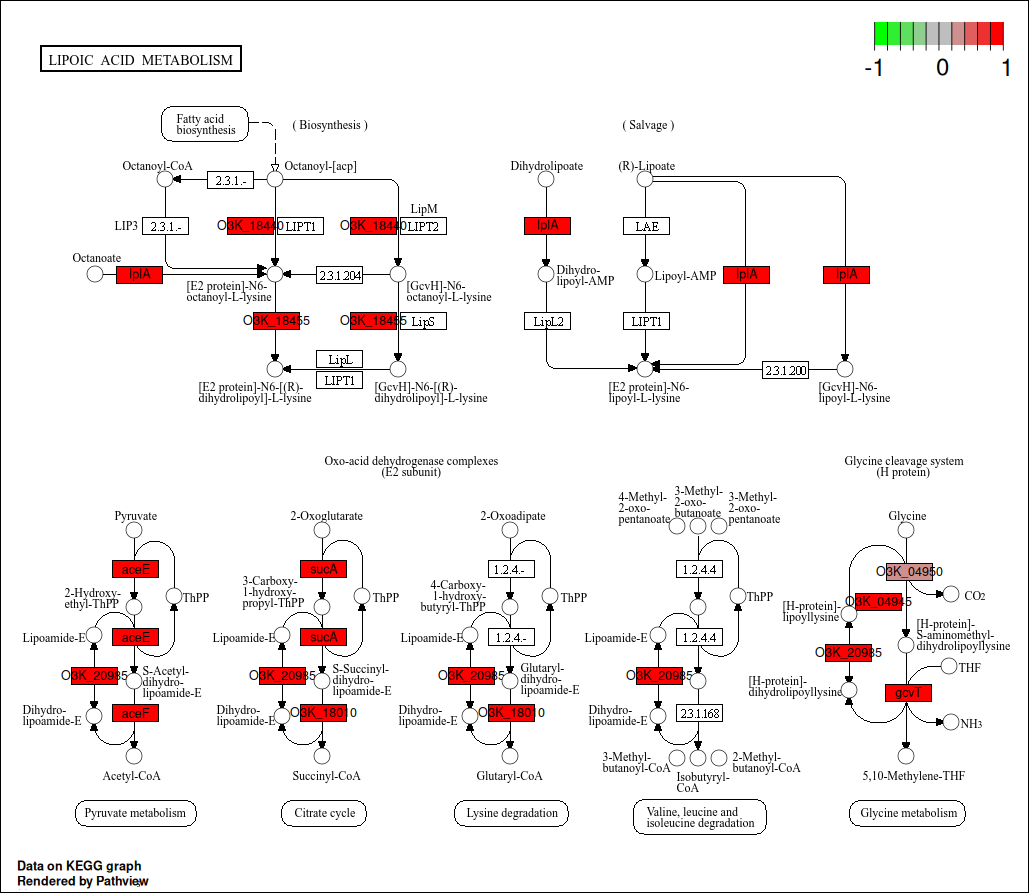


**(G)** The KEGG pathway “Lipoic acid metabolism” found activated in *E. coli* O104:H4 *rpoS* ATG > ATA in comparison to the wild type during transition. Colored genes are present in the strain. Gene expression values were mapped to the given gradient color scale.


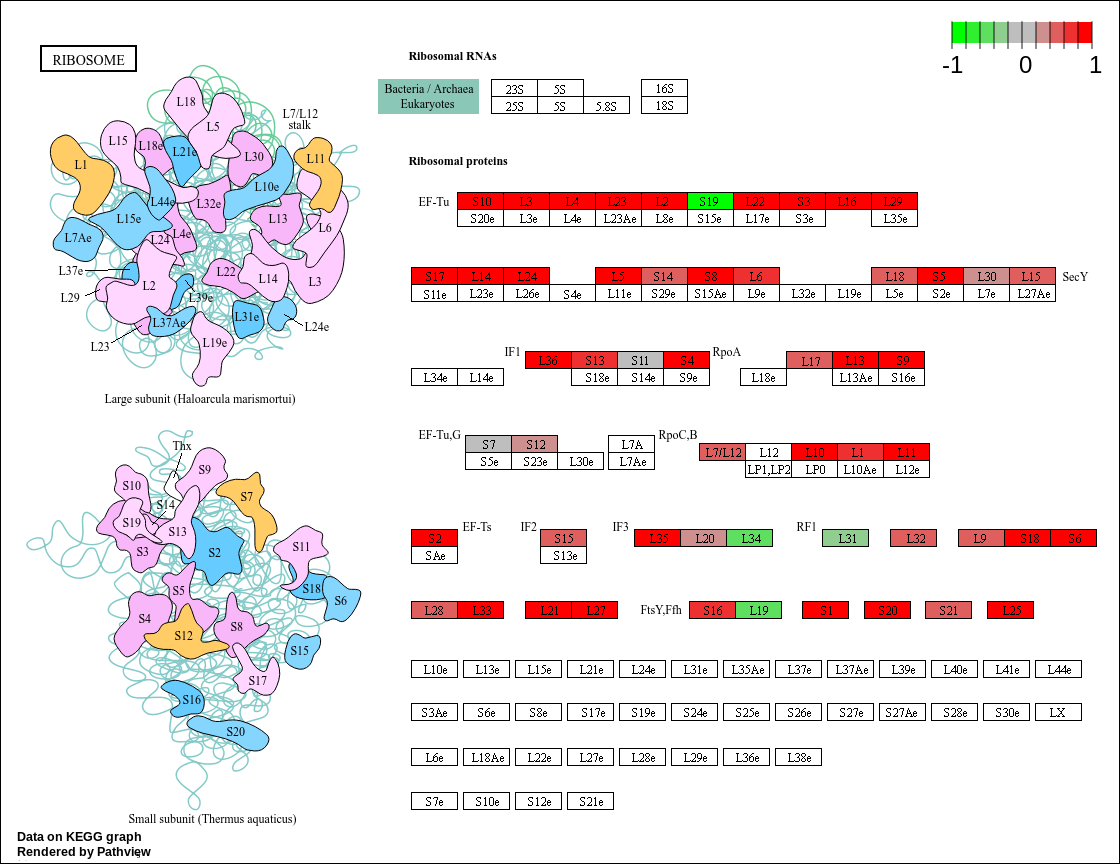


**(H)** The KEGG pathway “Ribosome” found activated in *E. coli* O104:H4 *rpoS* ATG > ATA in comparison to the wild type during transition. Genes encoding colored ribosomal protein are present in the strain. Gene expression values were mapped to the given gradient color scale.


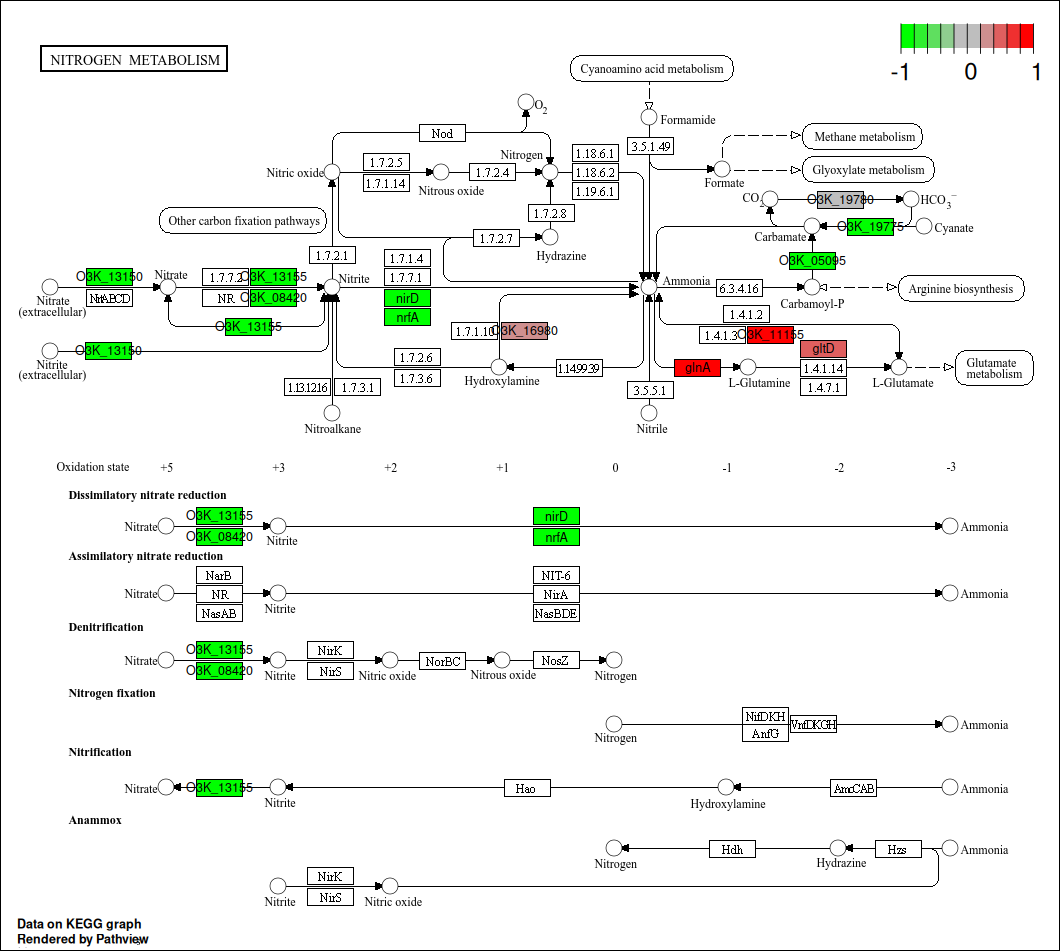


**(I)** The KEGG pathway “Nitrogen metabolism” found activated in *E. coli* O104:H4 *rpoS* ATG > ATA in comparison to the wild type during transition. Colored genes are present in the strain. Gene expression values were mapped to the given gradient color scale.


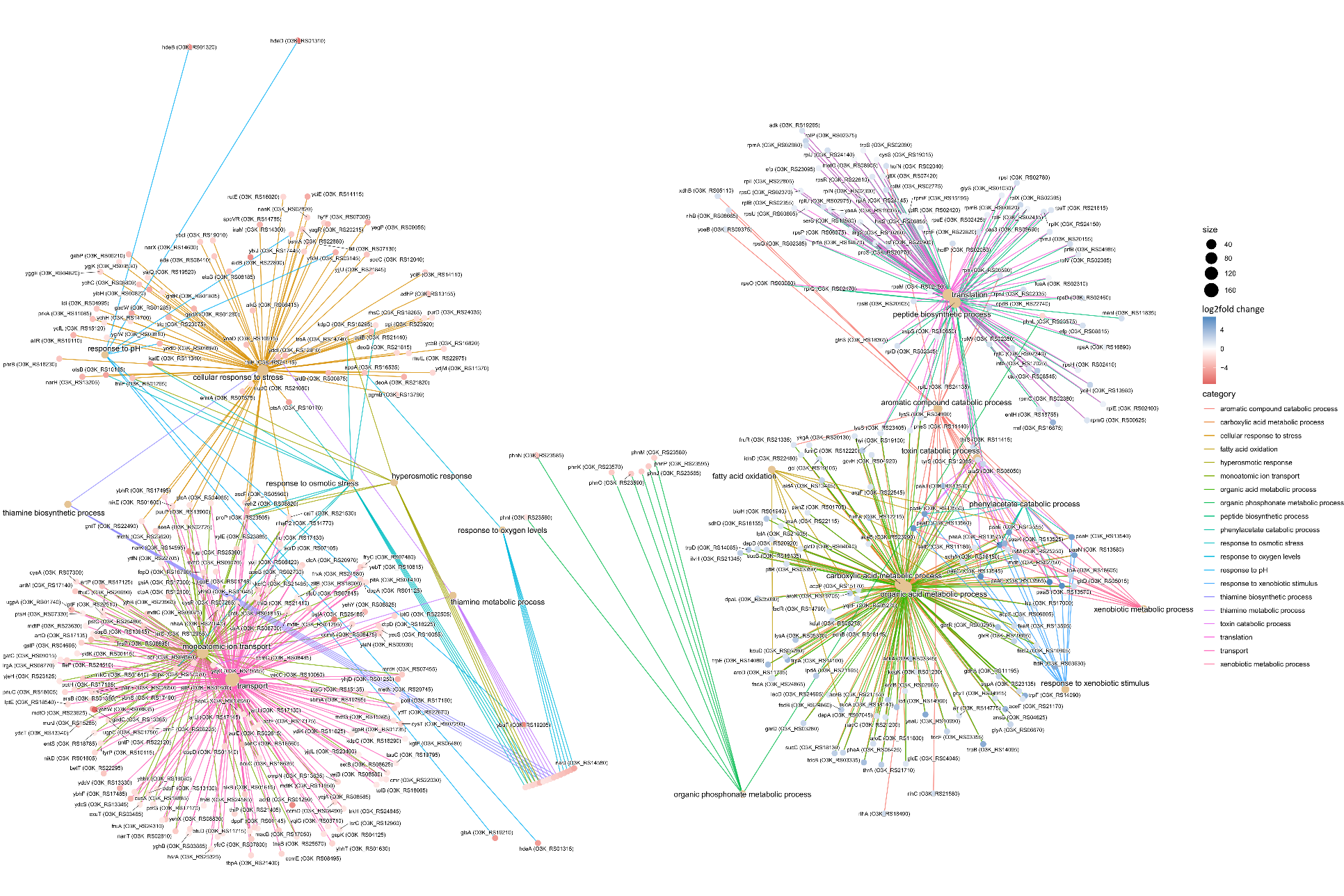


**Supplementary Figure 6. Cnet plot of enriched GO terms in *E. coli* O104:H4 *rpoS* ATG > ATA in comparison to the wild type in transition.**


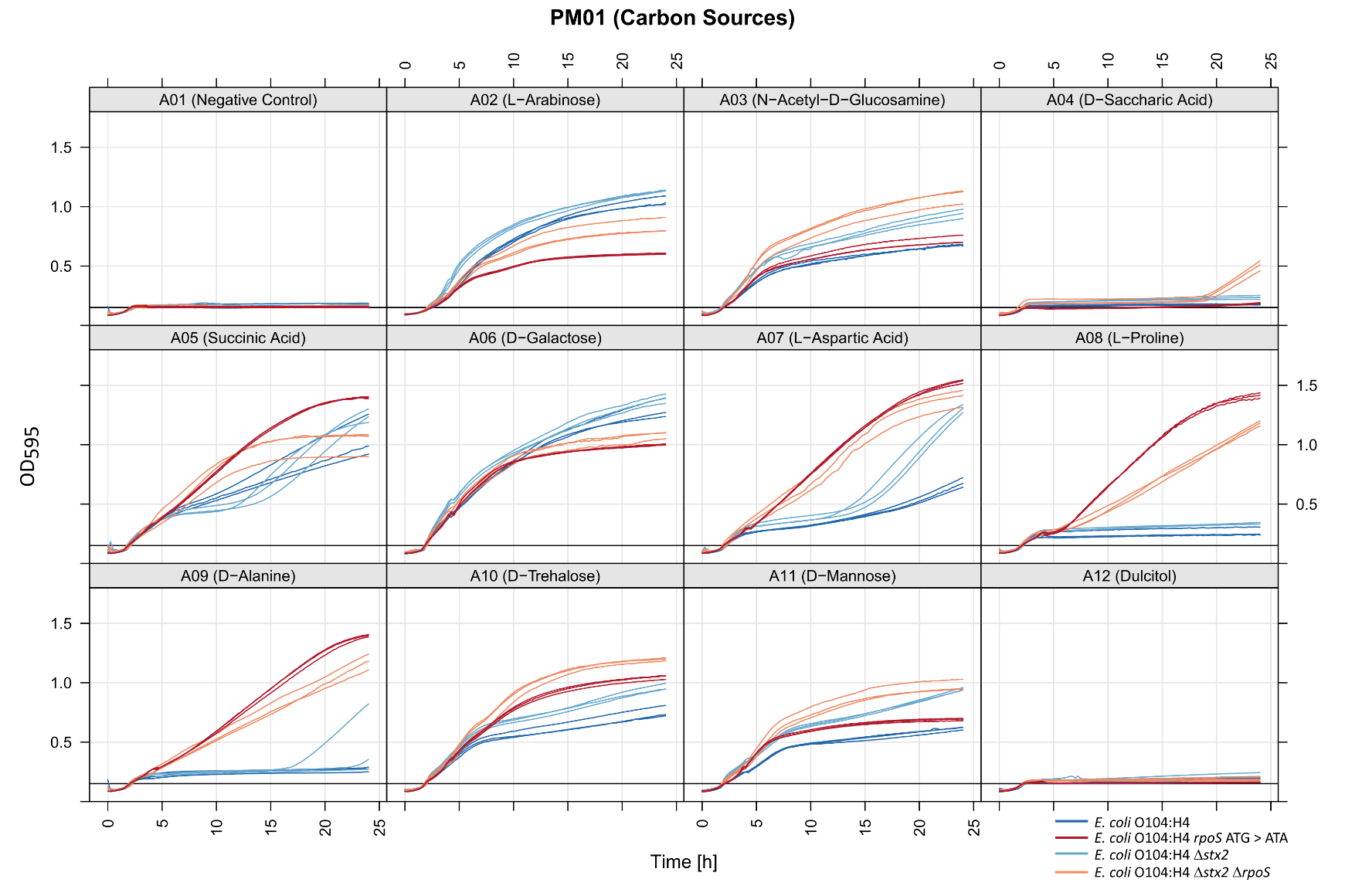


**Supplementary Figure 7. Kinetic measurements of the respiration potential of *E. coli* O104:H4 and the indicated derivative strains using BIOLOG PM1 MicroPlate^TM^ Carbon Sources.** The graphs represent the change in OD_595_ over time of three biological replicates per strain. **A. Lane A1-A12.**


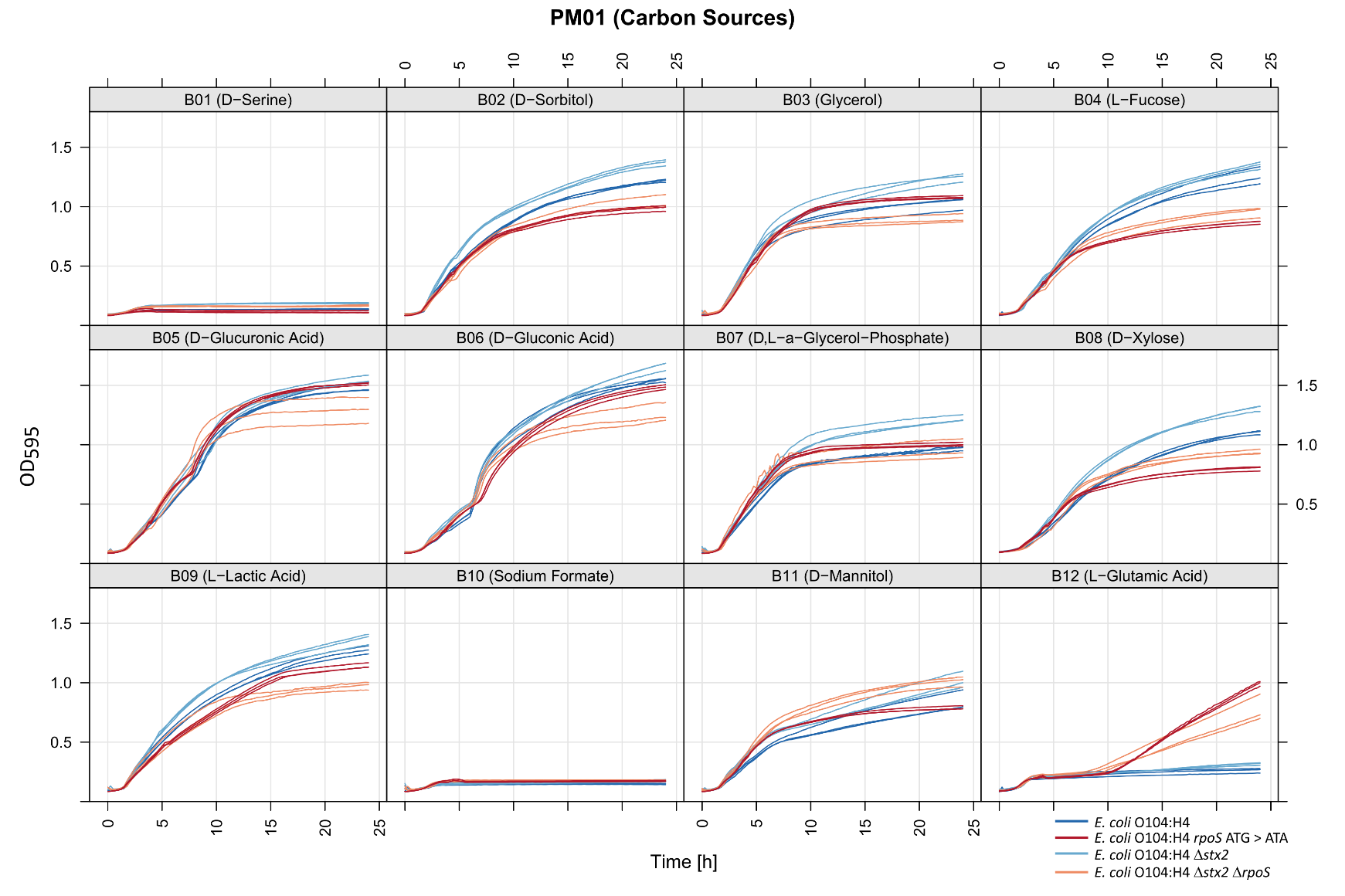


**B. Lane B1-B12.**


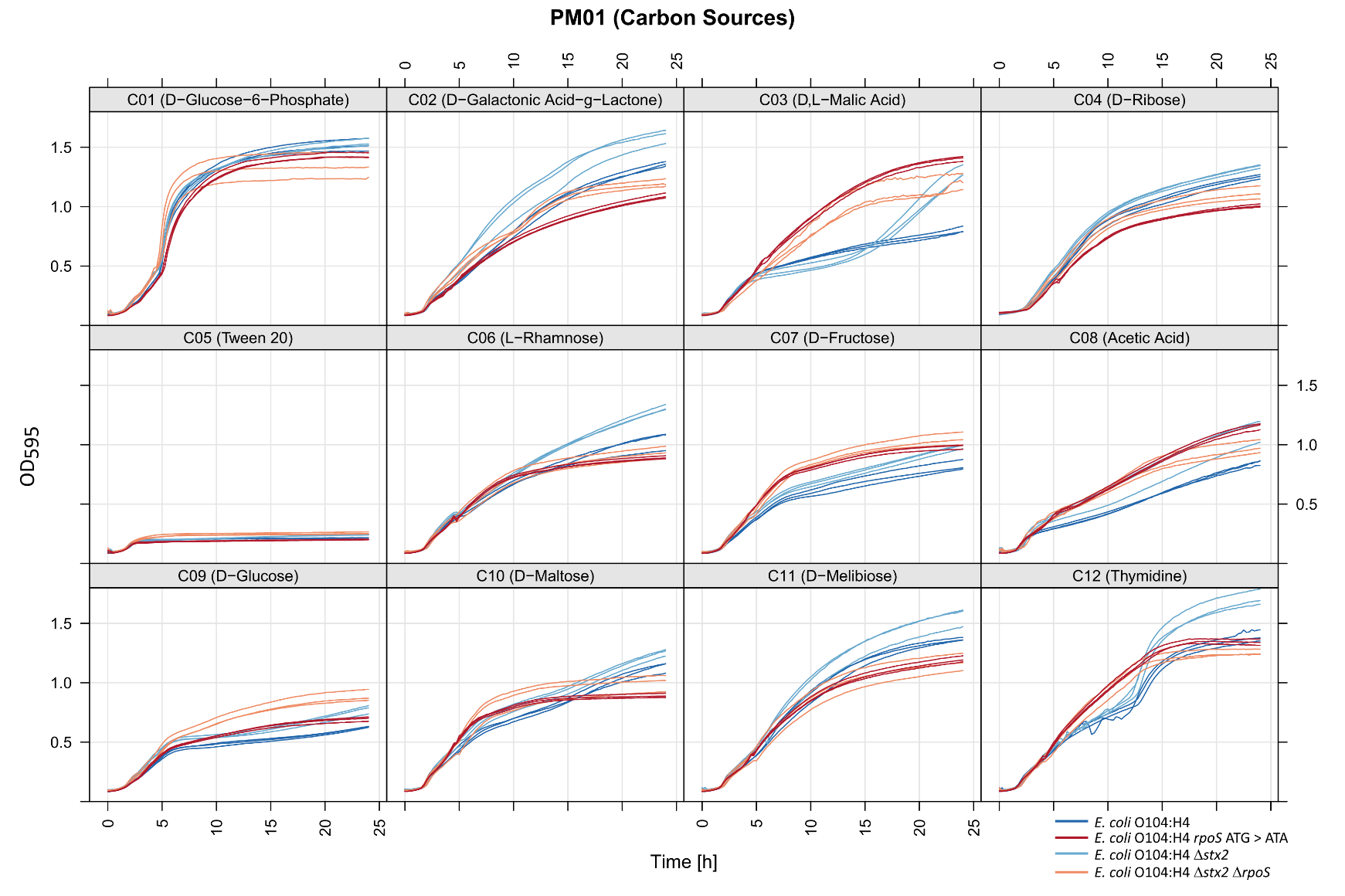


**C. Lane C1-C12.**


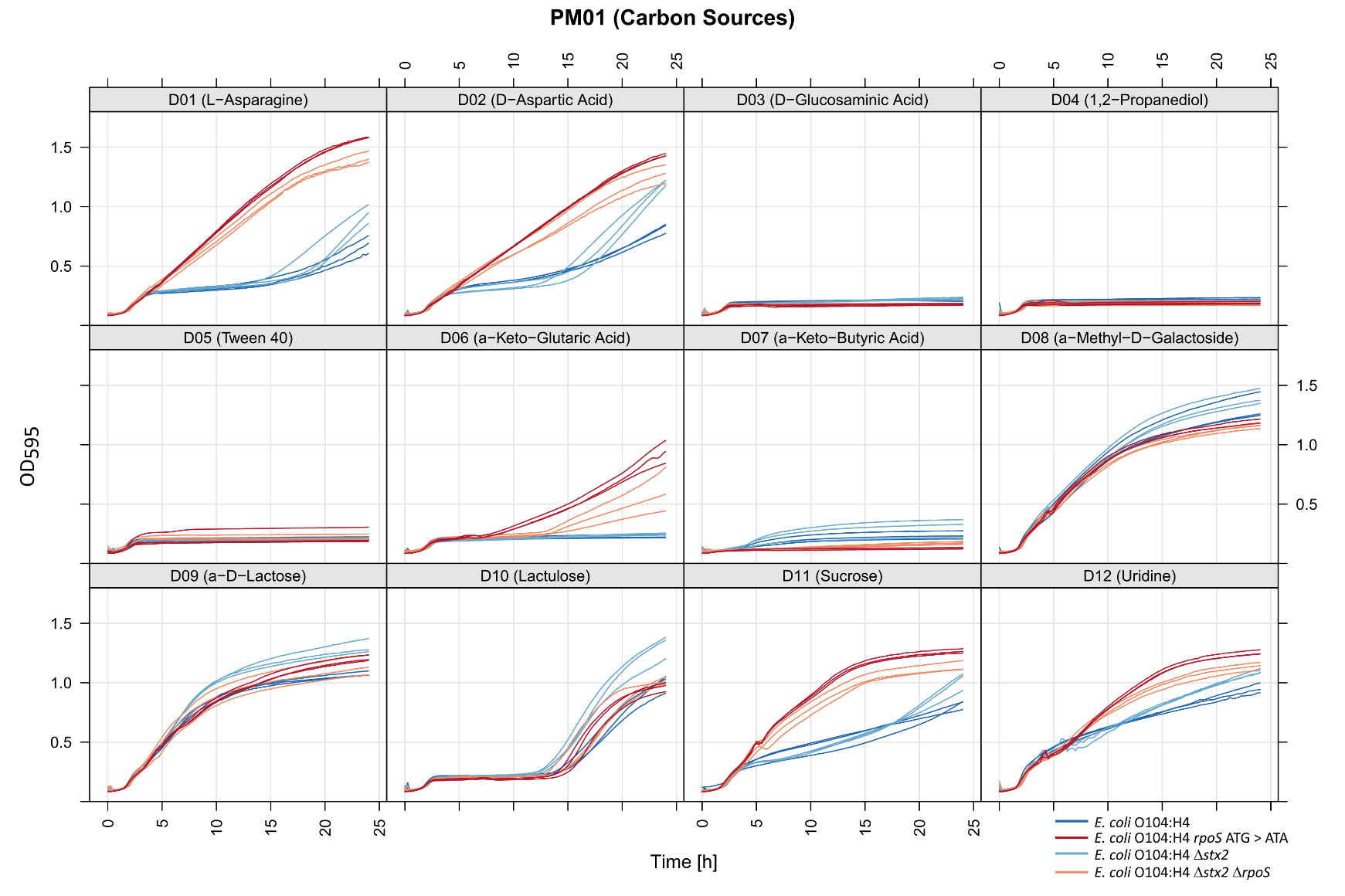


**D. Lane D1-D12.**

**
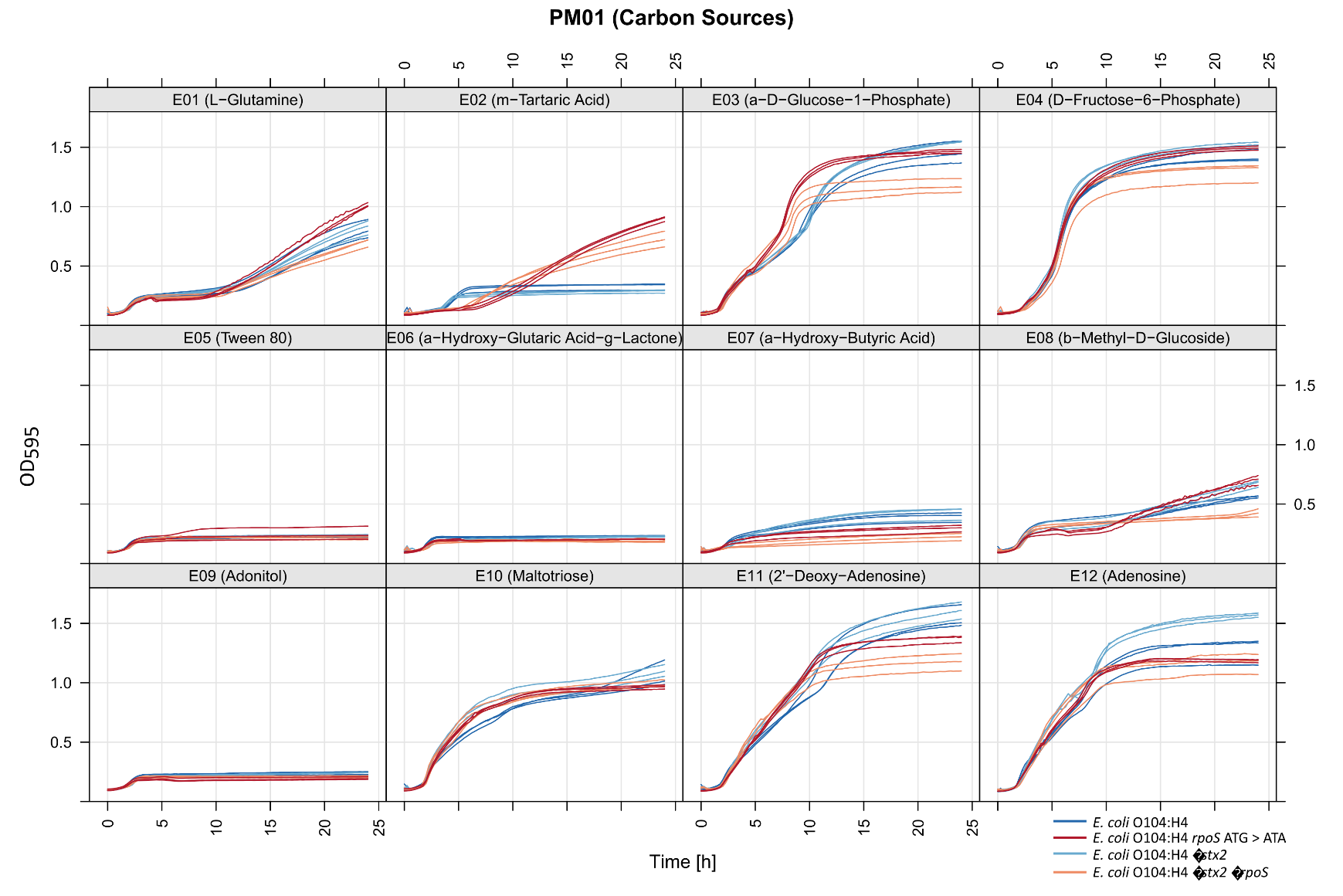
**

**E. Lane E1-E12.**


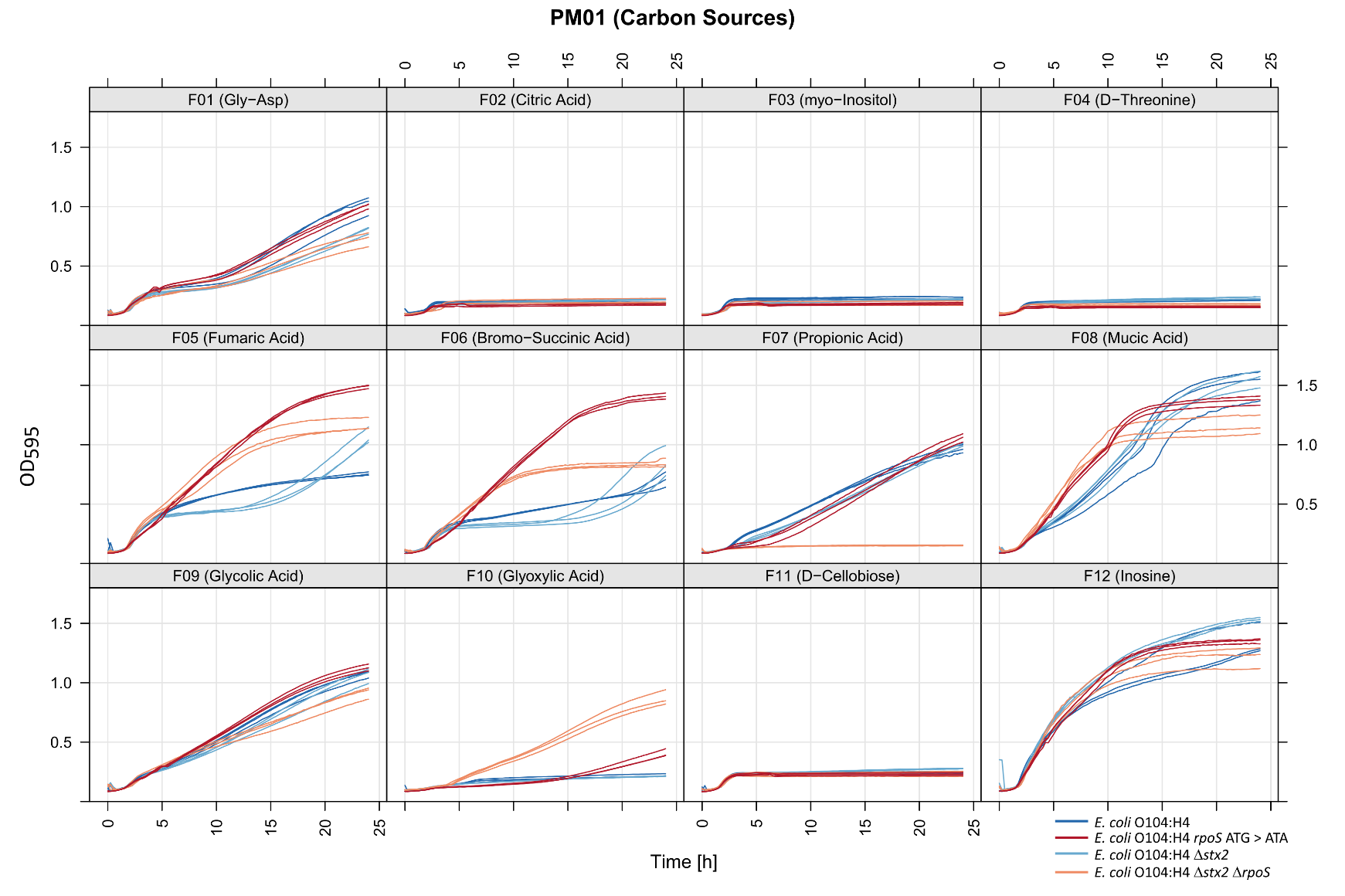


**F. Lane F1-F12.**


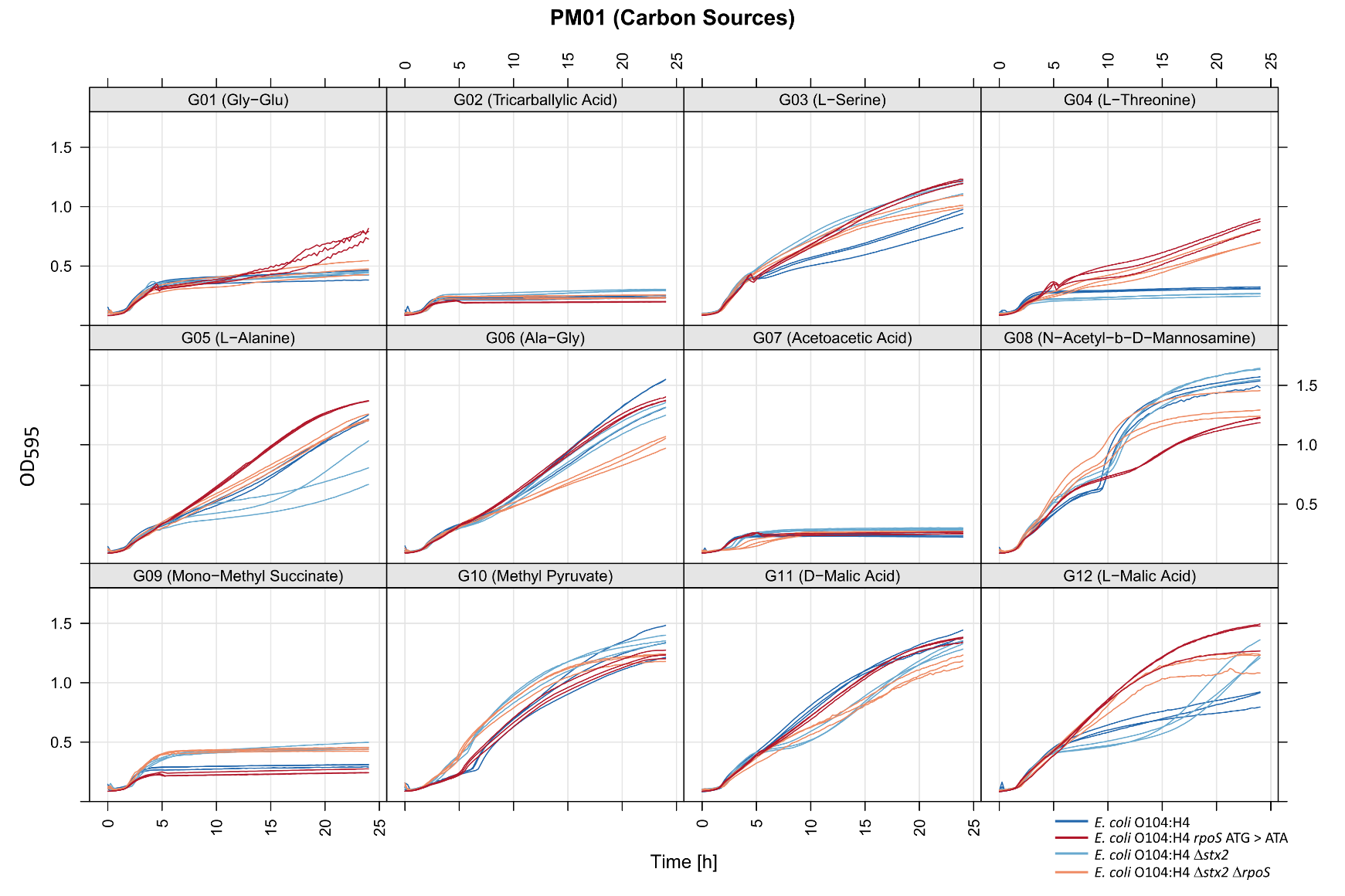


**G. Lane G1-G12.**


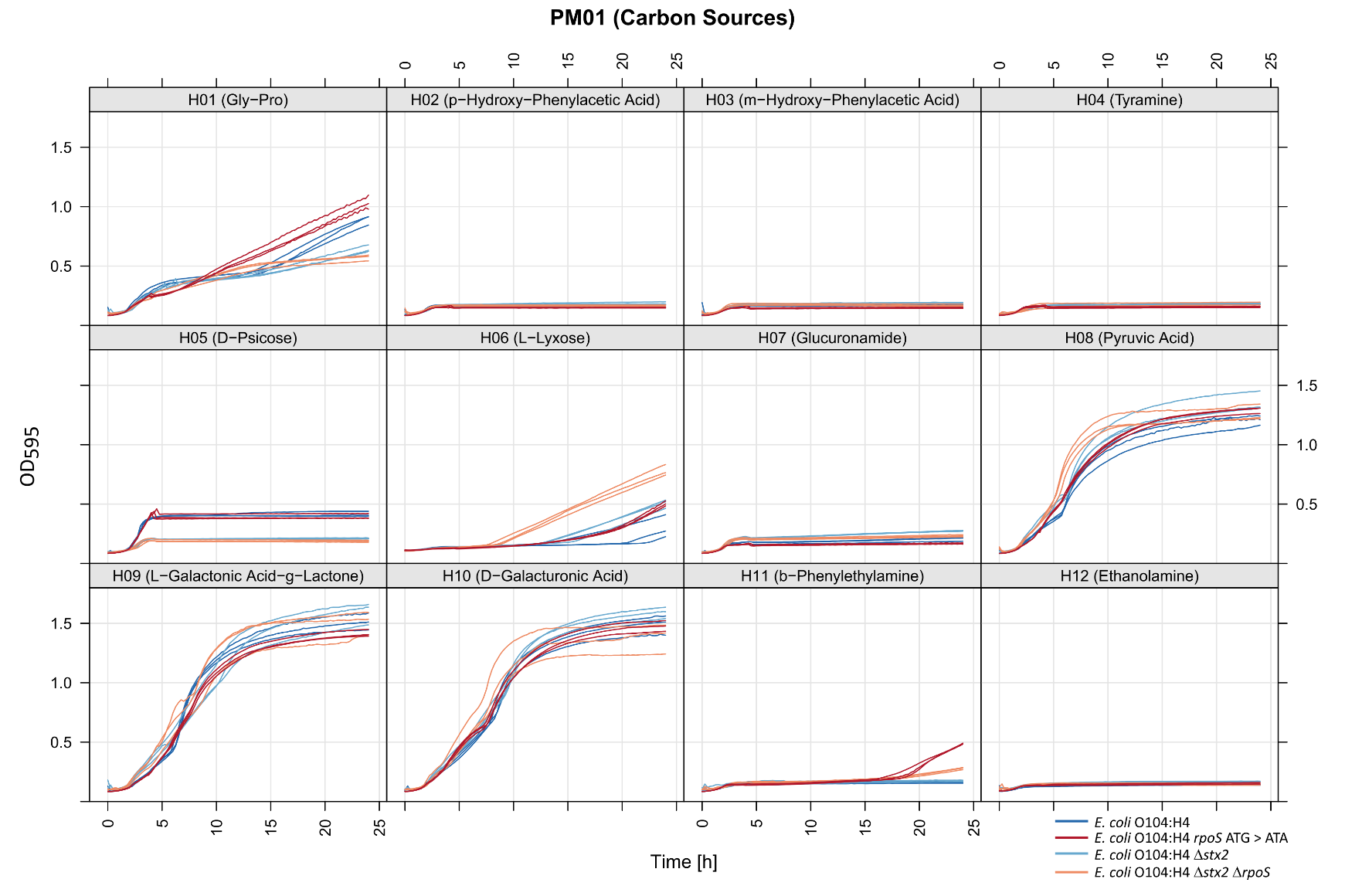


**H. Lane H1-H12.**


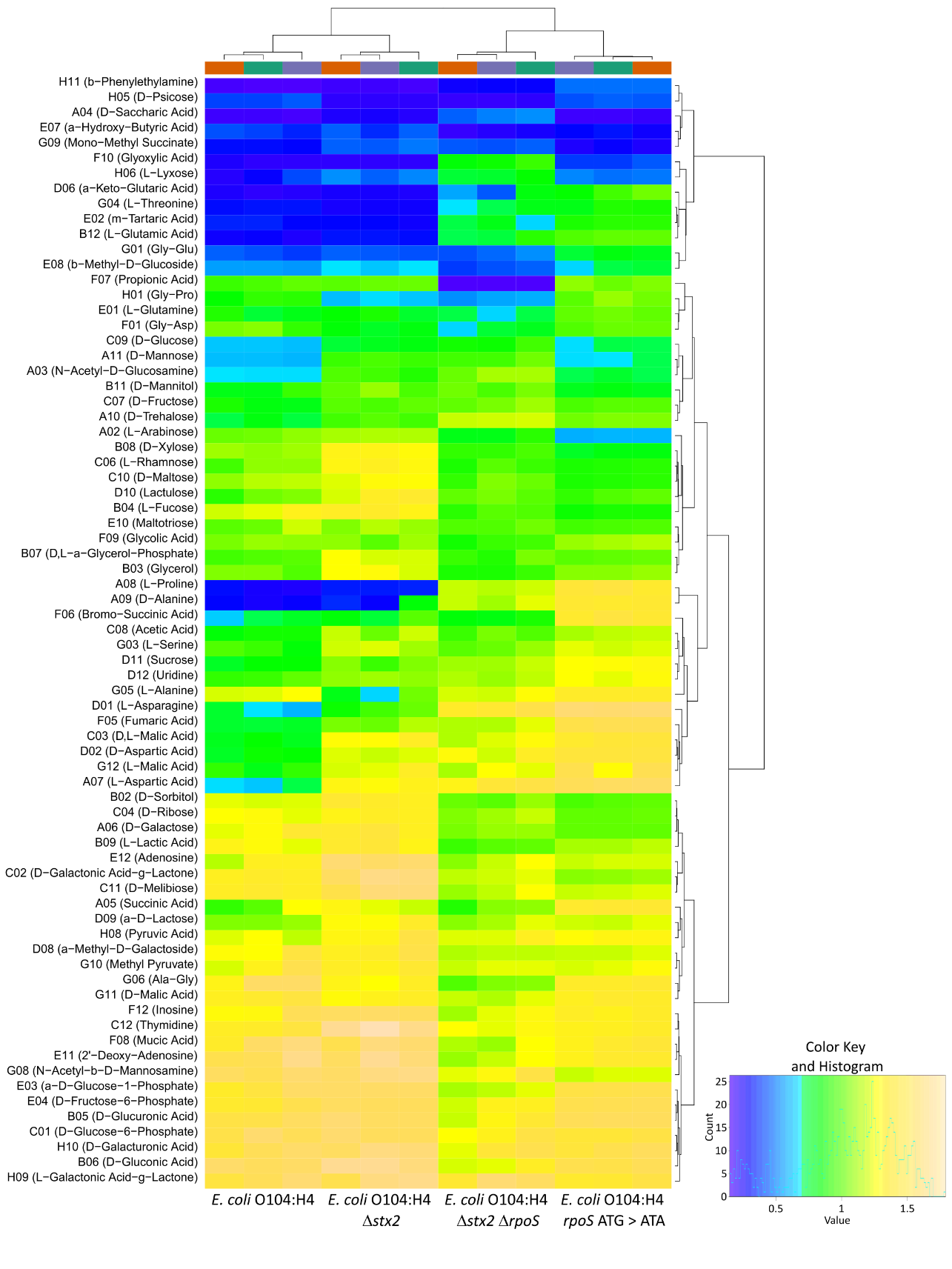
**Supplementary Figure 8. Heatmap of the respiration potential of all *E. coli* O104:H4 strains analyzed with the BIOLOG PM1.** Only substrates, which were assimilated at least by one of the strains are shown. Three biological replicates per strain were analyzed. The provided color key shows the degree of respiration (based on A; maximum curve height). The tree on top of the map shows the relationship between the analyzed samples and the tree on the side shows the relationship between the substrates.**
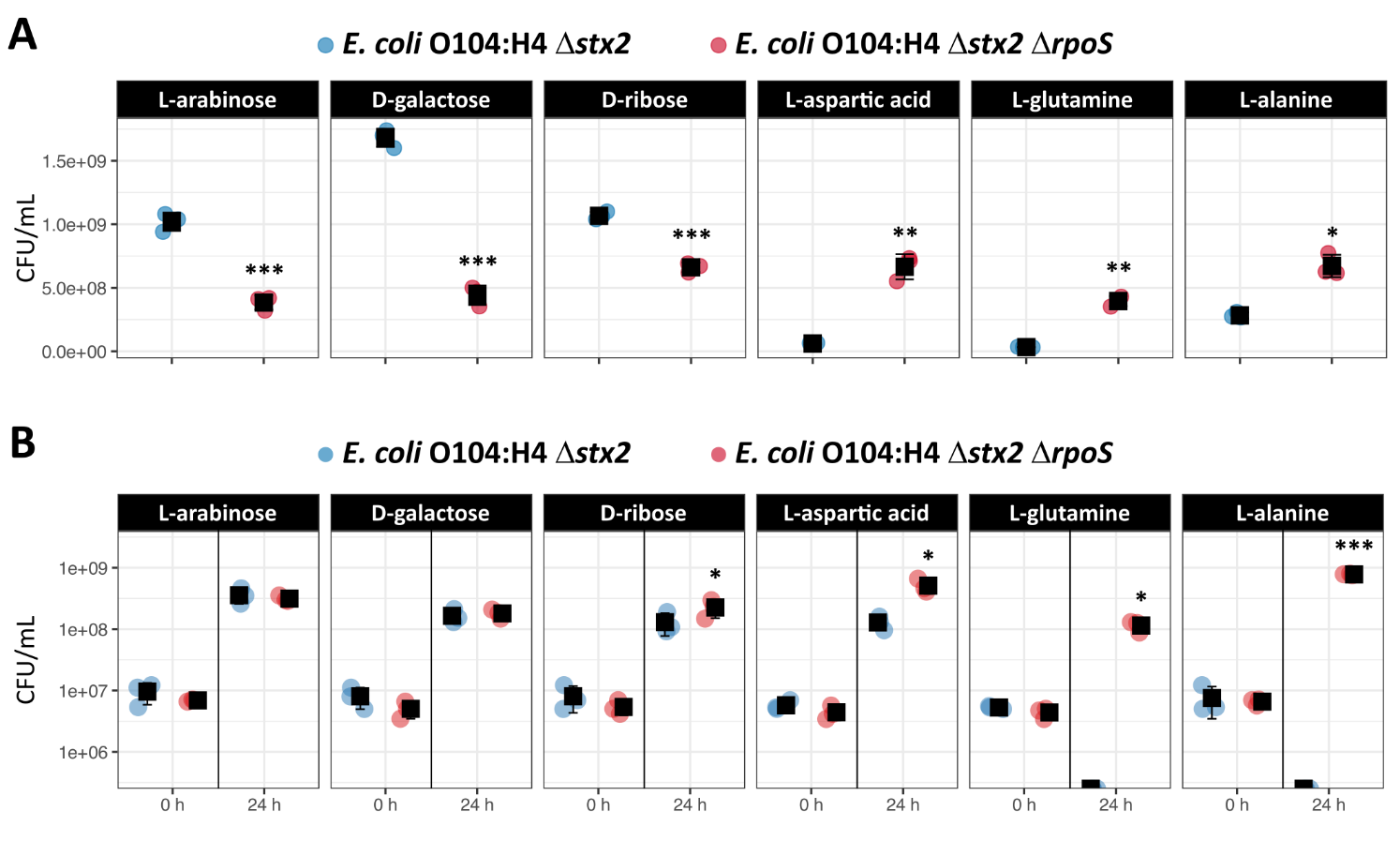
**

**Supplementary Figure 9.** **Growth experiments with *E. coli* O104:H4 Δ*stx2* and Δ*stx2* Δ*rpoS* in M9 medium supplemented with a single carbon source*.* (A) Singe culture experiments.** Final colony forming units per mL (CFU/mL) counts after 24 h of incubation during growth experiments presented in Figure 7A are given. Welch Two Sample T-test was used to assess the difference between samples. **(B) Co-culture experiments.** CFU/mL counts at inoculation (0 h) and at the end of the experiment (24 h). *E. coli* O104:H4 Δ*stx2* was not detected after 24 h in the presence of L-glutamine and L-alanine**.** Paired T-test was used to assess the difference between samples. Graphs were created using R package ggplot2 (circles = three biological replicates per bacterial strain, squares = mean values, error bars = standard deviations; * p < 0.05, ** p < 0.01, *** p < 0.001) and final figures were created with Inkscape.

## Supplementary Tables

**Supplementary Table 1. List of Biolog PM1 substrates assimilated by *E. coli* O104:H4 wild type and *rpoS* ATG > ATA.** Only substrates assimilated by at least one of the strains are shown. The substrate lane and names, substrate categories, strain with higher maximum curve height (A) and p value (adjusted for multiple comparisons) are given. The statistical analysis was performed using the opm package and the opm_mcp function with Turkey multiple comparison of means. The significant codes are given as follows: p < 0.001***, p < 0.01**, p < 0.05*.

| **Substrate** | **Substrate category** | **Strain with higher maximum curve height (A)** | **p value** |
| --- | --- | --- | --- |
| A02 (L−Arabinose) | Sugar | wild type | < 1e-05 *** |
| A03 (N−Acetyl−D−Glucosamine) | Sugar derivative |  | 1 |
| A07 (L−Aspartic Acid) | Amino acid | *rpoS* ATG > ATA | < 1e-05 *** |
| A06 (D−Galactose) | Sugar | wild type | < 1e-05 *** |
| A08 (L−Proline) | Amino acid | *rpoS* ATG > ATA | < 1e-05 *** |
| A09 (D−Alanine) | Amino acid | *rpoS* ATG > ATA | < 1e-05 *** |
| B12 (L−Glutamic Acid) | Amino acid | *rpoS* ATG > ATA | < 1e-05 *** |
| D01 (L−Asparagine) | Amino acid | *rpoS* ATG > ATA | < 1e-05 *** |
| A11 (D−Mannose) | Sugar |  | 0.963598 |
| B02 (D−Sorbitol) | Sugar derivative | wild type | < 1e-05 *** |
| B03 (Glycerol) | Sugar derivative |  | 1 |
| B04 (L−Fucose) | Sugar | wild type | < 1e-05 *** |
| B05 (D−Glucuronic Acid) | Sugar derivative |  | 1 |
| B06 (D−Gluconic Acid) | Sugar derivative |  | 0.999597 |
| B07 (D,L−a−Glycerol−Phosphate) | Sugar derivative |  | 1 |
| B08 (D−Xylose) | Sugar | wild type | < 1e-05 *** |
| A05 (Succinic Acid) | Organic acid (dicarboxylic) | *rpoS* ATG > ATA | < 1e-05 *** |
| B11 (D−Mannitol) | Sugar derivative |  | 0.999994 |
| D02 (D−Aspartic Acid) | Amino acid | *rpoS* ATG > ATA | < 1e-05 *** |
| C01 (D−Glucose−6−Phosphate) | Sugar derivative |  | 0.754387 |
| C02 (D−Galactonic Acid−g−Lactone) | Other | wild type | < 1e-05 *** |
| E01 (L−Glutamine) | Amino acid | *rpoS* ATG > ATA | < 1e-05 *** |
| C04 (D−Ribose) | Sugar | wild type | < 1e-05 *** |
| C06 (L−Rhamnose) | Sugar | wild type | 0.004170 ** |
| G03 (L−Serine) | Amino acid | *rpoS* ATG > ATA | < 1e-05 *** |
| G04 (L−Threonine) | Amino acid | *rpoS* ATG > ATA | < 1e-05 *** |
| C09 (D−Glucose) | Sugar |  | 0.996741 |
| C10 (D−Maltose) | Sugar | wild type | < 1e-05 *** |
| C11 (D−Melibiose) | Sugar | wild type | 0.000343 *** |
| C12 (Thymidine) | Nucleotide |  | 1 |
| G05 (L−Alanine) | Amino acid | *rpoS* ATG > ATA | 0.007116 ** |
| **Substrate** | **Substrate category** | **Strain with higher maximum curve height (A)** | **p value** |
| B09 (L−Lactic Acid) | Organic acid (carboxylic) | wild type | 0.021985 * |
| C03 (D,L−Malic Acid) | Organic acid (dicarboxylic) | *rpoS* ATG > ATA | < 1e-05 *** |
| D08 (a−Methyl−D−Galactoside) | Sugar derivative |  | 0.056614 |
| C08 (Acetic Acid) | Organic acid (carboxylic) | *rpoS* ATG >ATA | < 1e-05 *** |
| D10 (Lactulose) | Sugar |  | 1 |
| D06 (a−Keto−Glutaric Acid) | Organic acid (dicarboxylic) | *rpoS* ATG > ATA | < 1e-05 *** |
| E02 (m−Tartaric Acid) | Organic acid (dicarboxylic) | *rpoS* ATG > ATA | < 1e-05 *** |
| E07 (a−Hydroxy−Butyric Acid) | Organic acid (carboxylic) |  | 0.481936 |
| F05 (Fumaric Acid) | Organic acid (dicarboxylic) | *rpoS* ATG > ATA | < 1e-05 *** |
| E03 (a−D−Glucose−1−Phosphate) | Sugar derivative |  | 1 |
| E04 (D−Fructose−6−Phosphate) | Sugar derivative |  | 0.992784 |
| F06 (Bromo−Succinic Acid) | Organic acid (dicarboxylic) | *rpoS* ATG > ATA | < 1e-05 *** |
| F07 (Propionic Acid) | Organic acid (carboxylic) |  | 0.557694 |
| E10 (Maltotriose) | Sugar |  | 0.436398 |
| E11 (2'−Deoxy−Adenosine) | Nucleoside | wild type | 0.000109 *** |
| E12 (Adenosine) | Nucleoside |  | 0.848578 |
| F01 (Gly−Asp) | Dipeptide |  | 1 |
| F09 (Glycolic Acid) | Organic acid (carboxylic) |  | 0.999999 |
| G01 (Gly−Glu) | Dipeptide | *rpoS* ATG > ATA | 1e-05 *** |
| F10 (Glyoxylic Acid) | Organic acid (carboxylic) | *rpoS* ATG > ATA | 2.75e-05 *** |
| F08 (Mucic Acid) | Sugar derivative | wild type | 0.012404 * |
| G11 (D−Malic Acid) | Organic acid (dicarboxylic) |  | 1 |
| H01 (Gly−Pro) | Dipeptide | *rpoS* ATG > ATA | 0.005253 ** |
| F12 (Inosine) | Nucleoside |  | 1 |
| D12 (Uridine) | Nucleotide | *rpoS* ATG > ATA | < 1e-05 *** |
| H11 (b−Phenylethylamine) | Other | *rpoS* ATG > ATA | < 1e-05 *** |
| A10 (D−Trehalose) | Sugar | *rpoS* ATG > ATA | < 1e-05 *** |
| C07 (D−Fructose) | Sugar | *rpoS* ATG > ATA | 0.001315 ** |
| G06 (Ala−Gly) | Dipeptide |  | 0.709846 |
| G08 (N−Acetyl−b−D−Mannosamine) | Sugar derivative | wild type | < 1e-05 *** |
| G10 (Methyl Pyruvate) | Other |  | 0.220694 |
| G12 (L−Malic Acid) | Organic acid (dicarboxylic) | *rpoS* ATG > ATA | < 1e-05 *** |
| D09 (a−D−Lactose) | Sugar | *rpoS* ATG > ATA | 0.028042 * |
| D11 (Sucrose) | Sugar | *rpoS* ATG > ATA | < 1e-05 *** |
| H05 (D−Psicose) | Sugar |  | 1 |
| H06 (L−Lyxose) | Sugar | *rpoS* ATG > ATA | < 1e-05 *** |
| H08 (Pyruvic Acid) | Organic acid (carboxylic) |  | 0.898408 |
| H09 (L−Galactonic Acid−g−Lactone) | Other |  | 0.427517 |
| H10 (D−Galacturonic Acid) | Sugar derivative |  | 1 |
| E08 (b−Methyl−D−Glucoside) | Sugar derivative | *rpoS* ATG > ATA | 0.009955 ** |

**Supplementary Table 2. List of Biolog PM1 substrates assimilated by *E. coli* O104:H4 Δ*stx2* and Δ*stx2* Δ*rpoS*.** Only substrates assimilated at least by one of the strains are shown. The substrate lane and names, substrate categories, strain with higher maximum curve height (A) and p value (adjusted for multiple comparisons) are given. The statistical analysis of A or AUC was performed using the opm package and the opm_mcp function with Turkey multiple comparison of means. The significant codes are given as follows: p < 0.001***, p < 0.01**, p < 0.05*. C4-dicarboxilates with which *E. coli* O104:H4 Δ*stx2* Δ*rpoS* has bigger AUC but not A are marked in bold.

| **Substrate** | **Substrate Category** | **Strain with higher maximum curve height (A)** | **p value (A)** | **Strain with bigger area under the curve (AUC)** | **p value (AUC)** |
| --- | --- | --- | --- | --- | --- |
| A02 (L−Arabinose) | Sugar | Δ*stx2* | < 1e-05 *** | Δ*stx2* | < 1e-05 *** |
| A03 (N−Acetyl−D−Glucosamine) | Sugar derivative |  | 0.089424 | Δ*stx2* Δ*rpoS* | 0.006173 ** |
| A04 (D−Saccharic Acid) | Sugar derivative | Δ*stx2* Δ*rpoS* | < 1e-05 *** |  | 1 |
| **A05 (Succinic Acid)** | **Organic acid (dicarboxylic)** | **Δ*stx2*** | **0.000227 ***** | **Δ*stx2* Δ*rpoS*** | **0.000957 ***** |
| A06 (D−Galactose) | Sugar | Δ*stx2* | < 1e-05 *** | Δ*stx2* | 9.11e-05 *** |
| **A07 (L−Aspartic Acid)** | **Amino acid** |  | **0.987669** | **Δ*stx2* Δ*rpoS*** | **< 1e-05 ***** |
| A08 (L−Proline) | Amino acid | Δ*stx2* Δ*rpoS* | < 1e-05 *** | Δ*stx2* Δ*rpoS* | < 1e-05 *** |
| A09 (D−Alanine) | Amino acid | Δ*stx2* Δ*rpoS* | < 1e-05 *** | Δ*stx2* Δ*rpoS* | < 1e-05 *** |
| A10 (D−Trehalose) | Sugar | Δ*stx2* Δ*rpoS* | 4.85e-05 *** | Δ*stx2* Δ*rpoS* | < 1e-05 *** |
| A11 (D−Mannose) | Sugar |  | 1 |  | 0.258998 |
| B02 (D−Sorbitol) | Sugar derivative | Δ*stx2* | < 1e-05 *** | Δ*stx2* | < 1e-05 *** |
| B03 (Glycerol) | Sugar derivative | Δ*stx2* | < 1e-05 *** | Δ*stx2* | < 1e-05 *** |
| B04 (L−Fucose) | Sugar | Δ*stx2* | < 1e-05 *** | Δ*stx2* | < 1e-05 *** |
| B05 (D−Glucuronic Acid) | Sugar derivative | Δ*stx2* | < 1e-05 *** |  | 0.090128 . |
| B06 (D−Gluconic Acid) | Sugar derivative | Δ*stx2* | < 1e-05 *** | Δ*stx2* | < 1e-05 *** |
| B07 (D,L−a−Glycerol−Phosphate) | Sugar derivative | Δ*stx2* | < 1e-05 *** | Δ*stx2* | 2.90e-05 *** |
| B08 (D−Xylose) | Sugar | Δ*stx2* | `< 1e-05 *** | Δ*stx2* | < 1e-05 *** |
| B09 (L−Lactic Acid) | Organic acid (carboxylic) | Δ*stx2* | < 1e-05 *** | Δ*stx2* | 1e-05 *** |
| B11 (D−Mannitol) | Sugar derivative |  | 1 |  | 0.938908 |
| B12 (L−Glutamic Acid) | Amino acid | Δ*stx2* Δ*rpoS* | < 1e-05 *** | Δ*stx2* Δ*rpoS* | < 1e-05 *** |
| C01 (D−Glucose−6−Phosphate) | Sugar derivative | Δ*stx2* | 0.003018 ** |  | 0.903307 |
| C02 (D−Galactonic Acid−g−Lactone) | Other | Δ*stx2* | < 1e-05 *** | Δ*stx2* | < 1e-05 *** |
| **Substrate** | **Substrate Category** | **Strain with higher maximum curve height (A)** | **p value (A)** | **Strain with bigger area under the curve (AUC)** | **p value (AUC)** |
| **C03 (D,L−Malic Acid)** | **Organic acid (dicarboxylic)** |  | **0.999482** | **Δ*stx2* Δ*rpoS*** | **< 1e-05 ***** |
| C04 (D−Ribose) | Sugar | Δ*stx2* | 0.000155 *** | Δ*stx2* | 0.010538 * |
| C06 (L−Rhamnose) | Sugar | Δ*stx2* | < 1e-05 *** | Δ*stx2* | 0.000455 *** |
| C07 (D−Fructose) | Sugar |  | 1 | Δ*stx2* Δ*rpoS* | 0.004468 ** |
| C08 (Acetic Acid) | Organic acid (carboxylic) |  | 0.105562 |  | 1 |
|  |  |  |  |  |  |
| C09 (D−Glucose) | Sugar |  | 0.784358 | Δ*stx2* Δ*rpoS* | 0.009587 ** |
| C10 (D−Maltose) | Sugar | Δ*stx2* | < 1e-05 *** |  | 1 |
| C11 (D−Melibiose) | Sugar | Δ*stx2* | < 1e-05 *** | Δ*stx2* | < 1e-05 *** |
| C12 (Thymidine) | Nucleotide | Δ*stx2* | < 1e-05 *** | Δ*stx2* | 0.019249 * |
| D01 (L−Asparagine) | Amino acid | Δ*stx2* Δ*rpoS* | < 1e-05 *** | Δ*stx2* Δ*rpoS* | < 1e-05 *** |
| **D02 (D−Aspartic Acid)** | **Amino acid** |  | **0.999995** | **Δ*stx2* Δ*rpoS*** | **< 1e-05 ***** |
| D06 (a−Keto−Glutaric Acid) | Organic acid (dicarboxylic) | Δ*stx2* Δ*rpoS* | < 1e-05 *** | Δ*stx2* Δ*rpoS* | 0.027502 * |
| D08 (a−Methyl−D−Galactoside) | Sugar derivative | Δ*stx2* | 1.50e-05 *** | Δ*stx2* | 0.001114 ** |
| D09 (a−D−Lactose) | Sugar | Δ*stx2* | 0.045682 * | Δ*stx2* | 0.008696 ** |
| D10 (Lactulose) | Sugar | Δ*stx2* | < 1e-05 *** |  | 0.060515 . |
| D11 (Sucrose) | Sugar |  | 0.680909 | Δ*stx2* Δ*rpoS* | < 1e-05 *** |
| D12 (Uridine) | Nucleotide |  | 1 |  | 0.056724 . |
| E01 (L−Glutamine) | Amino acid |  | 0.452049 |  | 0.999922 |
| E02 (m−Tartaric Acid) | Organic acid (dicarboxylic) | Δ*stx2* Δ*rpoS* | < 1e-05 *** | Δ*stx2* Δ*rpoS* | < 1e-05 *** |
| E03 (a−D−Glucose−1−Phosphate) | Sugar derivative | Δ*stx2* | < 1e-05 *** | Δ*stx2* | 0.001647 ** |
| E04 (D−Fructose−6−Phosphate) | Sugar derivative | Δ*stx2* | 4.51e-05 *** | Δ*stx2* | 1.13e-05 *** |
| E07 (a−Hydroxy−Butyric Acid) | Organic acid (carboxylic) | Δ*stx2* | 0.001258 ** | Δ*stx2* | < 1e-05 *** |
| E08 (b−Methyl−D−Glucoside) | Sugar derivative | Δ*stx2* | 1.16e-05 *** |  | 0.465915 |
| E10 (Maltotriose) | Sugar |  | 0.764811 |  | 0.999999 |
| E11 (2'−Deoxy−Adenosine) | Nucleoside | Δ*stx2* | < 1e-05 *** | Δ*stx2* | < 1e-05 *** |
| E12 (Adenosine) | Nucleoside | Δ*stx2* | < 1e-05 *** | Δ*stx2* | < 1e-05 *** |
| F01 (Gly−Asp) | Dipeptide |  | 0.99993 |  | 1 |
| **F05 (Fumaric Acid)** | **Organic acid (dicarboxylic)** |  | **0.935309** | **Δ*stx2* Δ*rpoS*** | **< 1e-05 ***** |
| **F06 (Bromo−Succinic Acid)** | **Organic acid (dicarboxylic)** |  | **1** | **Δ*stx2* Δ*rpoS*** | **< 1e-05 ***** |
| **Substrate** | **Substrate Category** | **Strain with higher maximum curve height (A)** | **p value (A)** | **Strain with bigger area under the curve (AUC)** | **p value (AUC)** |
| F07 (Propionic Acid) | Organic acid (carboxylic) | Δ*stx2* | < 1e-05 *** | Δ*stx2* | < 1e-05 *** |
| F08 (Mucic Acid) | Sugar derivative | Δ*stx2* | < 1e-05 *** |  | 1 |
| F09 (Glycolic Acid) | Organic acid (carboxylic) |  | 0.132895 |  | 1 |
| F10 (Glyoxylic Acid) | Organic acid (carboxylic) | Δ*stx2* Δ*rpoS* | < 1e-05 *** | Δ*stx2* Δ*rpoS* | < 1e-05 *** |
| F12 (Inosine) | Nucleoside | Δ*stx2* | < 1e-05 *** | Δ*stx2* | 0.000222 *** |
| G01 (Gly−Glu) | Dipeptide |  | 1 |  | 1 |
| G03 (L−Serine) | Amino acid |  | 0.145385 |  | 0.800272 |
| G04 (L−Threonine) | Amino acid | Δ*stx2* Δ*rpoS* | < 1e-05 *** | Δ*stx2* Δ*rpoS* | < 1e-05 *** |
| G05 (L−Alanine) | Amino acid | Δ*stx2* Δ*rpoS* | < 1e-05 *** | Δ*stx2* Δ*rpoS* | < 1e-05 *** |
| G06 (Ala−Gly) | Dipeptide | Δ*stx2* | < 1e-05 *** | Δ*stx2* | 0.004162 ** |
| G08 (N−Acetyl−b−D−Mannosamine) (N−Acetyl−b−D−Mannosamine) | Sugar derivative | Δ*stx2* | < 1e-05 *** |  | 0.714433 |
| G09 (Mono−Methyl Succinate) | Other |  | 1 |  | 1 |
| G10 (Methyl Pyruvate) | Other |  | 0.169565 |  | 0.999964 |
| G11 (D−Malic Acid) | Organic acid (dicarboxylic) |  | 0.266675 |  | 1 |
| **G12 (L−Malic Acid)** | **Organic acid (dicarboxylic)** |  | **0.999999** | **Δ*stx2* Δ*rpoS*** | **< 1e-05 ***** |
| H01 (Gly−Pro) | Dipeptide |  | 0.999983 |  | 1 |
| H06 (L−Lyxose) | Sugar | Δ*stx2* Δ*rpoS* | < 1e-05 *** | Δ*stx2* Δ*rpoS* | 0.000101 *** |
| H08 (Pyruvic Acid) | Organic acid (carboxylic) |  | 0.964266 |  | 1 |
| H09 (L−Galactonic Acid−g−Lactone) | Other |  | 0.998232 |  | 1 |
| H10 (D−Galacturonic Acid) | Sugar derivative | Δ*stx2* | 0.000617 *** |  | 0.990851 |

**Supplementary Table 3. Lag phase duration of *E. coli* O104:H4 Δ*stx2* and Δ*stx2* Δ*rpoS*** **growth in M9 medium supplemented with a single carbon source*.*** The lag phase was calculated using the growth curves provided in Figure 7A and the tangent method on a microbial lag phase duration calculator (<https://microbialgrowth.shinyapps.io/lag_calulator/>). If not otherwise indicated, the table presents the mean and standard deviation of three biological replicates. Welch Two Sample T-test was used to assess the difference between samples. The significant codes are given as follows: p < 0.01**, p < 0.05*.

| **Strain** | **Carbon source** | **Lag phase duration [h]** | **p value** |
| --- | --- | --- | --- |
| *E. coli* O104:H4 Δ*stx2* | L-arabinose | 3.6 ± 0.4 | 0.01956 * |
| *E. coli* O104:H4 Δ*stx2* Δ*rpoS* |  | 2.6 ± 0.3 |  |
| *E. coli* O104:H4 Δ*stx2* | D-galactose | 3.0 ± 0.2**^#^** | NA **^†^** |
| *E. coli* O104:H4 Δ*stx2* Δ*rpoS* |  | 2.1 ± 0.1 |  |
| *E. coli* O104:H4 Δ*stx2* | D-ribose | 5.8 ± 0.4 | 0.007673 ** |
| *E. coli* O104:H4 Δ*stx2* Δ*rpoS* |  | 3.9 ± 0.5 |  |
| *E. coli* O104:H4 Δ*stx2* | L-aspartic acid | 21.1 ± 1.4 | 0.001008 ** |
| *E. coli* O104:H4 Δ*stx2* Δ*rpoS* |  | 2.5 ± 0.4 |  |
| *E. coli* O104:H4 Δ*stx2* | L-glutamine | 2.3 ± 3.1 | 0.6232 |
| *E. coli* O104:H4 Δ*stx2* Δ*rpoS* |  | 3.4 ± 1.5 |  |
| *E. coli* O104:H4 Δ*stx2* | L-alanine | 1.5 ± 0.9**^#^** | NA **^†^** |
| *E. coli* O104:H4 Δ*stx2* Δ*rpoS* |  | 2.5 ± 0.4 |  |

**^#^**Mean and standard deviation of two biological replicates; lag phase duration of 0 was detected for one replicate and omitted from the analysis.

^†^No statistical analysis was performed due to small sample size (n < 3).
